# Enhancing the epidemiological surveillance of SARS-CoV-2 using Sanger sequencing to identify circulating variants and recombinants

**DOI:** 10.1007/s42770-024-01387-x

**Published:** 2024-05-28

**Authors:** Thaís Silva, Eneida Oliveira, Alana Oliveira, André Menezes, Wander de Jesus Jeremias, Rafaella FQ Grenfell, Rubens Lima do Monte-Neto, Marcelo A. Pascoal-Xavier, Marco A. Campos, Gabriel Fernandes, Pedro Alves

**Affiliations:** 1 Instituto René Rachou, Fundação Oswaldo Cruz, 1715, Augusto de Lima Avenue, Belo Horizonte, Minas Gerais 30190-002 Brazil; 2 Secretaria Municipal de Saúde, 2336, Afonso Pena Avenue, Belo Horizonte, Minas Gerais 30130-007 Brazil; 3 Department of Pharmacy, Federal University of Ouro Preto (UFOP), 27, Nine Street, Ouro Preto, Minas Gerais 35400-000 Brazil; 4 Department of Infectious Diseases, College of Veterinary Medicine, University of Georgia, 501 D.W. Brooks Drive, Athens, GA 30602 USA; 5 Department of Anatomic Pathology, College of Medicine, Federal University of Minas Gerais, 6627, Presidente Antônio Carlos Avenue, Belo Horizonte, Minas Gerais 31270-901 Brazil

**Keywords:** Sanger, SARS-CoV-2, Monitoring, Variants, Sequencing, Recombinants

## Abstract

Since the emergence of SARS-CoV-2 in December 2019, more than 12,000 mutations in the virus have been identified. These could cause changes in viral characteristics and directly impact global public health. The emergence of variants is a great concern due to the chance of increased transmissibility and infectivity. Sequencing for surveillance and monitoring circulating strains is extremely necessary as the early identification of new variants allows public health agencies to make faster and more effective decisions to contain the spread of the virus. In the present study, we identified circulating variants in samples collected in Belo Horizonte, Brazil, and detected a recombinant lineage using the Sanger method. The identification of lineages was done through gene amplification of SARS-CoV-2 by Reverse Transcription-Polymerase Chain Reaction (RT-PCR). By using these specific fragments, we were able to differentiate one variant of interest and five circulating variants of concern. We were also able to detect recombinants. Randomly selected samples were sequenced by either Sanger or Next Generation Sequencing (NGS). Our findings validate the effectiveness of Sanger sequencing as a powerful tool for monitoring variants. It is easy to perform and allows the analysis of a larger number of samples in countries that cannot afford NGS.

**Supplementary Information:**

The online version contains supplementary material available at 10.1007/s42770-024-01387-x.

## Introduction

Since the detection of Severe Acute Respiratory Syndrome Coronavirus 2 (SARS-CoV-2) in December 2019 in Wuhan, China, the virus has evolved. The observed changes in the SARS-CoV-2 genome may lead to new variants with the potential to change some viral characteristics, such as transmissibility, increased disease severity, vaccine escape or immune response, and an increase in mortality. The evolution of SARS-CoV-2 raised the reproductive number (R_0_) to a level above 5 for the Delta variant (B.1.617.2). In contrast, at the beginning of the pandemic, this number for the ancestral strain was estimated to be below 3 [1–3].

Estimates suggest that circulating SARS-CoV-2 lineages accumulate nucleotide mutations at a rate of about 1–2 per month, which is a high rate compared to other RNA viruses [4, 5]. Three nomenclature systems for naming and tracking SARS-CoV-2 variants are currently used: GISAID, Pango, and Nextstrain [6]. In addition to these three systems, the variants are also classified according to the criteria established by the World Health Organization (WHO). This classification system was updated on 15 March 2023 and classified the variants into variants of concern (VOC, using letters from the Greek alphabet), variants of interest (VOI), and variants under monitoring (VUM). The previous circulating VOCs were: Alpha (B.1.1.7), Beta (B.1.351), Gamma (P.1), Delta (B.1.617.2), and Omicron (B.1.1.529). Lambda (C.37) and Mu (B.1.621) were considered variants of interest. Although the variant Zeta (P.2) had previously been classified as a variant of interest, as of July 2021, it is no longer considered as such by the WHO. In May 2023, there was no VOC in circulation, but there were two VOIs (XBB.1.15 and XBB.1.16) and seven VUMs (BA.2.75, CH.1.1, BQ.1, XBB.*, XBB.1.9.1, XBB.1.9.2 and XBB.2.3) [7].

Given the sustained dissemination and evolution of SARS-CoV-2, new variants are expected to emerge. Therefore, methods to identify circulating and possible new variants must be implemented to mitigate and contain COVID-19 effectively. In addition, monitoring circulating variants provides essential information about changes in the epidemiological profile of COVID-19. Since the beginning of the pandemic, Whole Genome Sequencing (WGS) has been the main method performed worldwide through Next Generation Sequencing (NGS) method [8, 9]. However, these methods are excessively expensive as they demand consumables, infrastructure, and staff to conduct the tests and analyze the results. This makes them difficult to apply in mass testing, especially in low-income countries. Methods based on real-time PCR assays and Sanger technology have been described as important alternative methods [10–14].

In Brazil in 2020, the representativeness of complete genomes sequenced was insufficient to understand the dynamics of SARS-CoV-2 circulation in the country [15]. Data from GISAID's EpiCoV™ database showed that only 1,826 sequences had been submitted in 2020, which is relatively few considering the COVID-19 pandemic's impact on the world and the urgent need to identify circulating strains to reduce the spread of SARS-CoV-2 [16]. As of 2021, the representativeness of complete genomes has increased considerably. However, in a country of continental proportions, with many regional differences, there was a significant disparity in data availability between states [15]. In November 2020, Gamma (P.1) variant was first discovered in Brazil and it was considered a variant of concern in 2021. This is an example of the importance of improving whole genome sequencing [15]. One of the states most severely affected by the COVID-19 epidemic was Amazonas, where the Gamma variant (P.1) was first discovered. This variant was found in Brazilian travelers who had recently arrived in Japan [17]. Therefore, it was important to use alternatives to the NGS method to infer the circulation of the different variants of SARS-CoV-2 and quickly provide the data epidemiological surveillance services [11, 12, 18]. During the pandemic in Brazil, there was a great disparity in the adoption of restrictive measures, with divergences in the pattern of social distancing measures and in the time of their adoption in different states [19, 20]. In that way, together with the more restrictive measures adopted by the city of Belo Horizonte, variant tracking has become essential for controlling the pandemic, in addition to the important preparedness of the hospital system [21]. This study aimed to use and compare capillary electrophoresis DNA sequencing (Sanger sequencing) with NGS for monitoring and screening variants of SARS-CoV-2 in the city of Belo Horizonte, Minas Gerais, Brazil.

## Results

### Genomic analysis to identify and track circulating SARS-CoV-2 variants over time

**Fig. 1:**
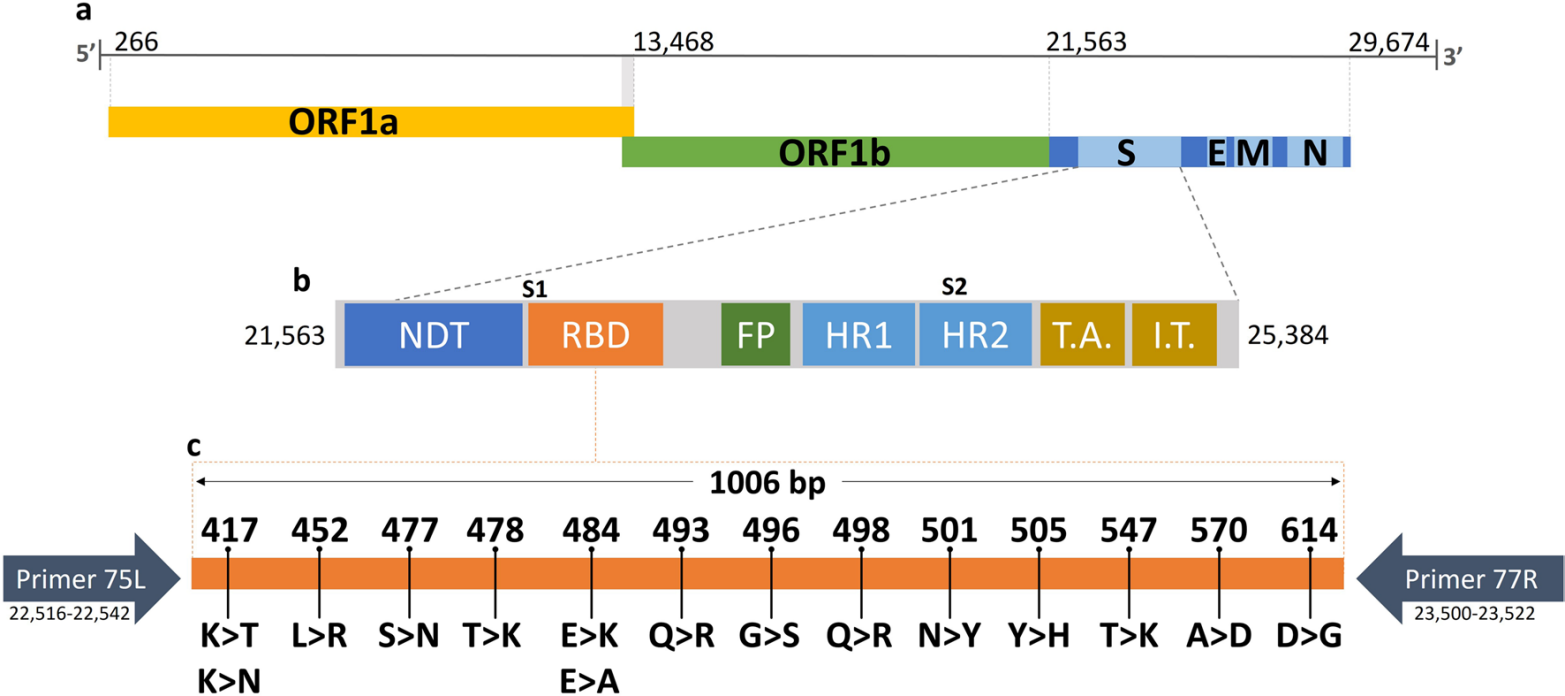
Scheme representing the SARS-CoV-2 genome. (**a**) and (**b**) represent the viral genome and the spike protein organization, respectively. (**c**) is the 1006 bp fragment amplified (in orange) with primers 75L and 77R, allowing the identification of mutations at positions 417, 452, 477, 478, 484, 493, 496, 498, 501, 505, 547, 570, and 614. The amino acid changes at each of the positions are shown in **c**

Sequences were generated from 317 out of 397 samples that met the quality criteria for Sanger sequencing. Those 317 sequences represent an overall success rate of 79.8% for identifying variants. Figure 1 shows the methodology employed to amplify a segment of the Spike protein encompassing variant-defining mutations between positions 417 and 614 to allow the identification of the circulating SARS-CoV-2 variants.

**Fig. 2:**
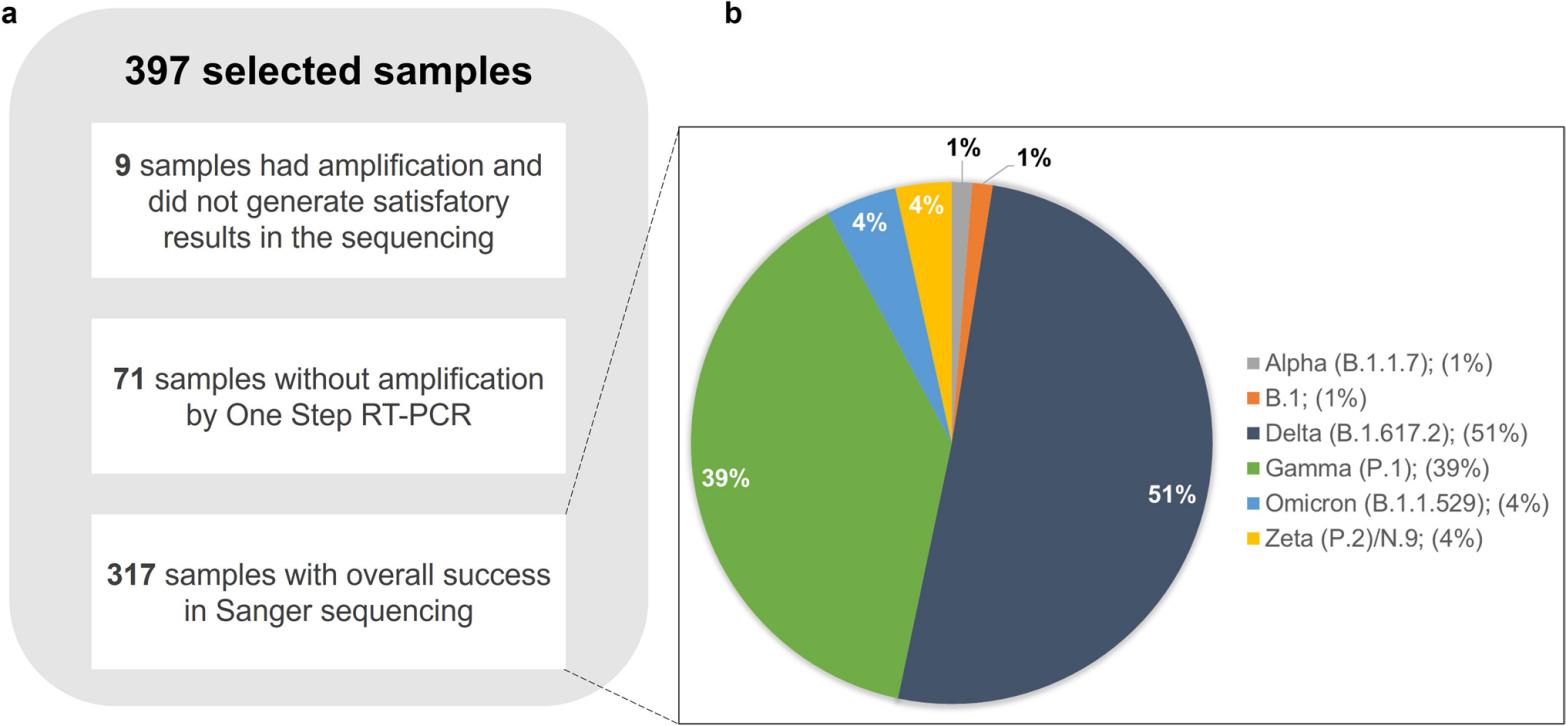
Diagram showing the efficiency of the identification of SARS-CoV2 variants using Sanger sequencing. (**a**) shows the amplification and sequencing results of the 397 samples selected for the study; (**b**) shows the percentage of variants found in the sequenced samples (n = 317)

We did not amplify DNA from 71 samples, representing 17.9% of the total. Nine (2.3%) had the fragment amplified and did not generate satisfactory results in the sequencing (Fig. 2a). Regarding the sensitivity of the Sanger method for identifying VOC and VOI variants through Spike gene fragment, 97.2% of the samples that had the fragment amplified were correctly sequenced after the amplification process. A correct sequencing result means that sequences in these samples exhibited Phred values greater than 20 (99% accuracy in base calling) in most of the bases in the amplified fragment, enabling the identification of variant-defining mutations. If we consider the failure to distinguish between Zeta and N.9 variants, this number drops to 93.9%. However, it is important to highlight that the method successfully identified all VOCs present up to the time of the study (Figure S1). The Delta variant (B.1.617.2) represented the highest number identified, followed by the Gamma variant (P.1) and Omicron variant (B.1.1.529). We also found the Zeta variant (P.2), which was considered a VOI until June 2021. It is important to highlight the emergence of Omicron. Shortly after its identification, its numbers were growing rapidly. It was also possible to identify the SARS-CoV-2 lineage B.1. (Fig. 2b).

**Fig. 3:**
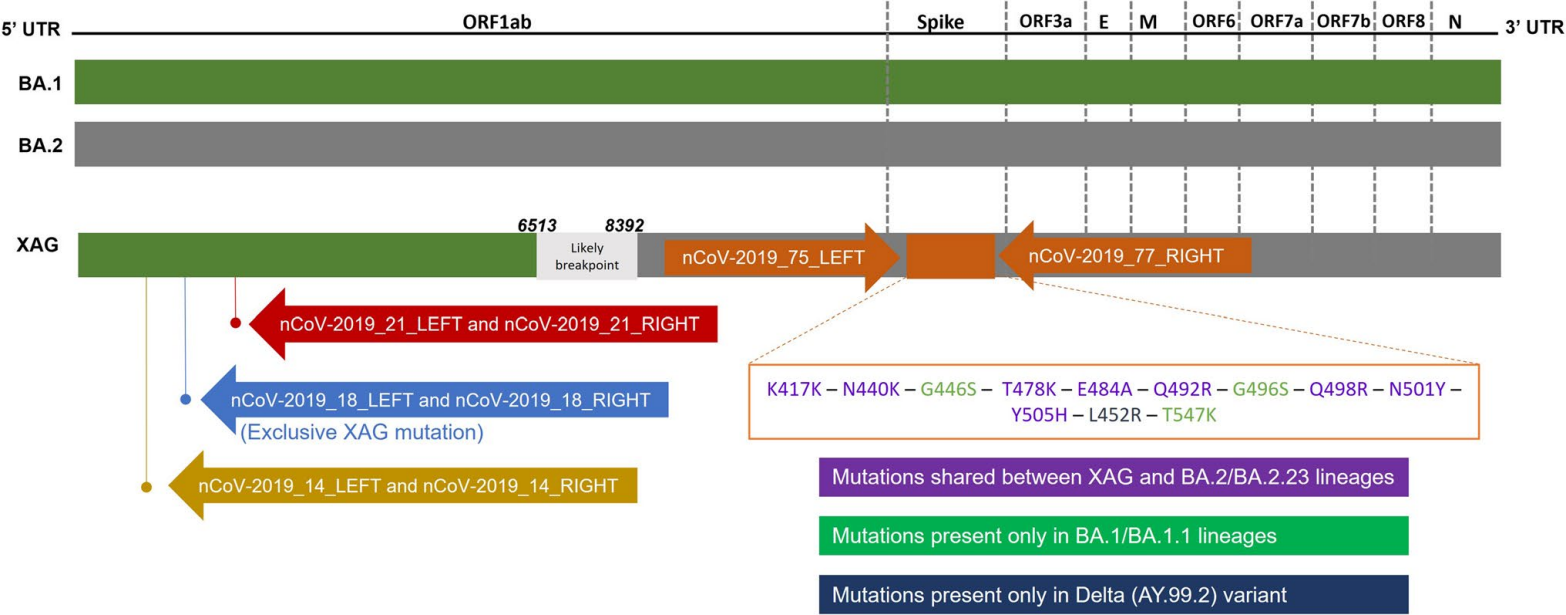
Schematic representation of the primers used for the detection of XAG recombinant, referring to BA.1. Four sets of primers were used to detect four exclusive XAG mutations: the set nCoV-2019_14 (highlighted in yellow) detected the mutations between 3992 and 4409 positions; set nCoV-2019_18 (highlighted in blue) detected the mutations between 5230 and 5643 positions, including a exclusive XAG mutation; the set nCoV-2019_21 (highlighted in red) detected the mutations between 6184 and 6582 positions positions, including a deletion in the 6512 position. In the portion referring to BA.2, the nCoV-2019_75_LEFT and nCoV-2019_77_RIGHT primers were used (highlighted in orange). The mutations highlighted in purple are shared between the BA.2/BA.2.23 lineages and the recombinant XAG, while the mutations highlighted in green are present only in the BA.1/BA.1.1 lineages. The mutations highlighted in blue are present only in the Delta variant.

Regarding the recombinant samples, supplementary Table 1 summarizes the mutations found in all the fragments amplified for XAG identification. To detect recombinants, sets of primers were used that amplify fragments in regions of ORF1ab that allow the distinction between the XAG variant and other variants such as the Delta and sublineages of Omicron (BA.1 and BA.2). The variant-defining mutations were identified employing sets of primers that amplify regions of ORF1a and the spike protein gene. The variant-defining mutations detected in the fragment amplified in the Spike gene region are shown in Fig. 1. In addition to this fragment, three sets of primers that amplify different regions of the ORF1a gene were added to identify the XAG recombinant lineage (Fig. 3).

**Fig. 4:**
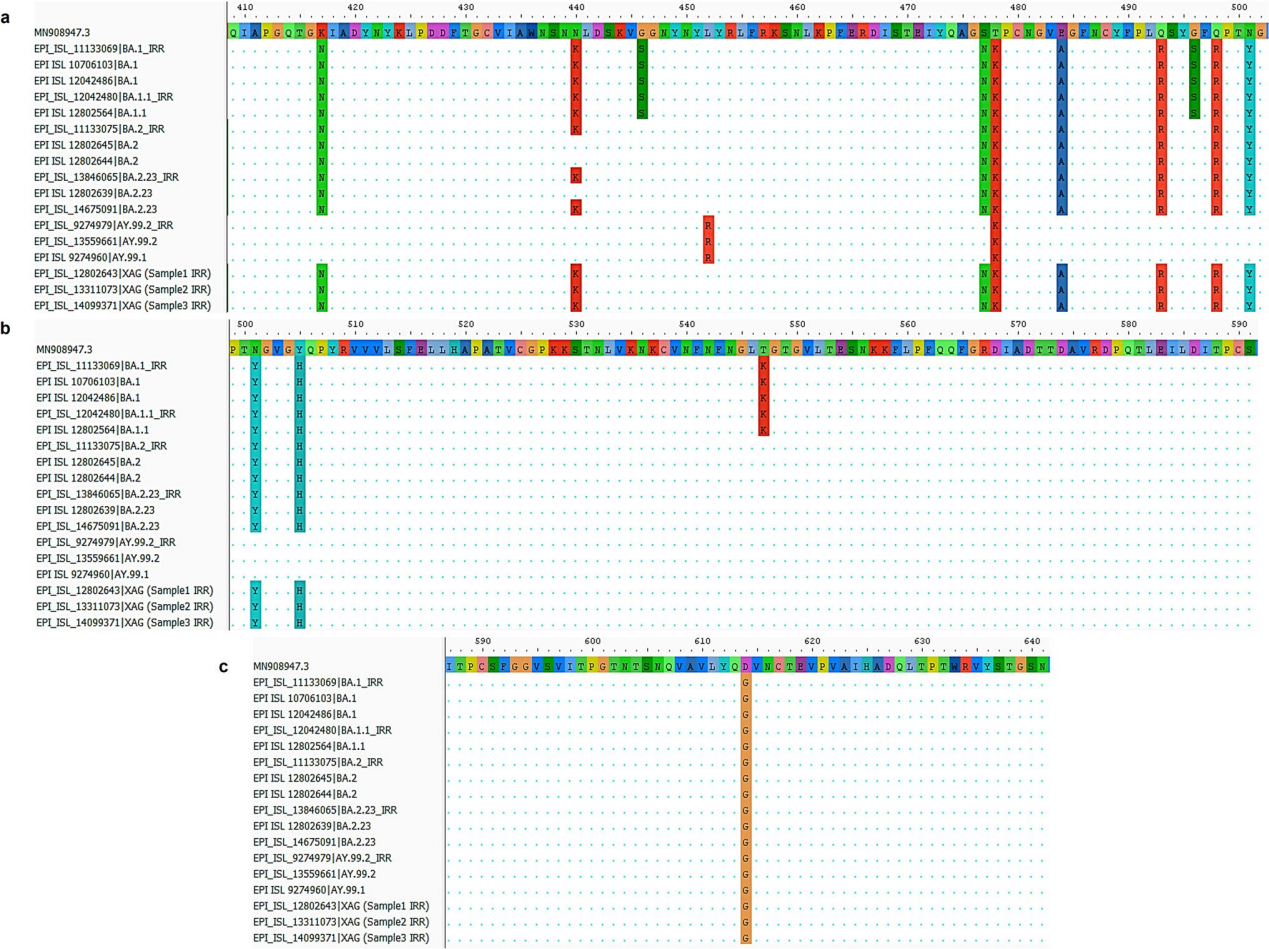
Alignment of the spike protein amplified fragment. Alignments were visualized using AliView software (version 1.28). (**a**) shows mutations from positions 410 to 500; (**b**) shows mutations from position 500 to 590 and (**c**) shows mutations from positions 590 to 640. MN908947.3: Severe Acute Respiratory Syndrome Coronavirus 2 isolate Wuhan-Hu-1, complete genome

Using the set of primers CoV-2019_75_LEFT and nCoV-2019_77_RIGHT, which amplifies a region in the Spike protein, we were able to distinguish between the BA.1/BA.1.1, Delta, and XAG lineages. Fourteen mutations occurred in this amplified region, of which three appear only in the BA.1/BA.1.1 sequences and one in the Delta variant sequence (AY.99.2) (Fig. 4). As expected, due to the genetic similarity between these two lineages in the amplified genomic region, all mutations identified in BA.2/BA.2.23 were also found in the sequences of the three recombinant XAG samples. These results demonstrate the potential use of this tool in other BA.1/BA.2 recombinants.

**Fig. 5:**
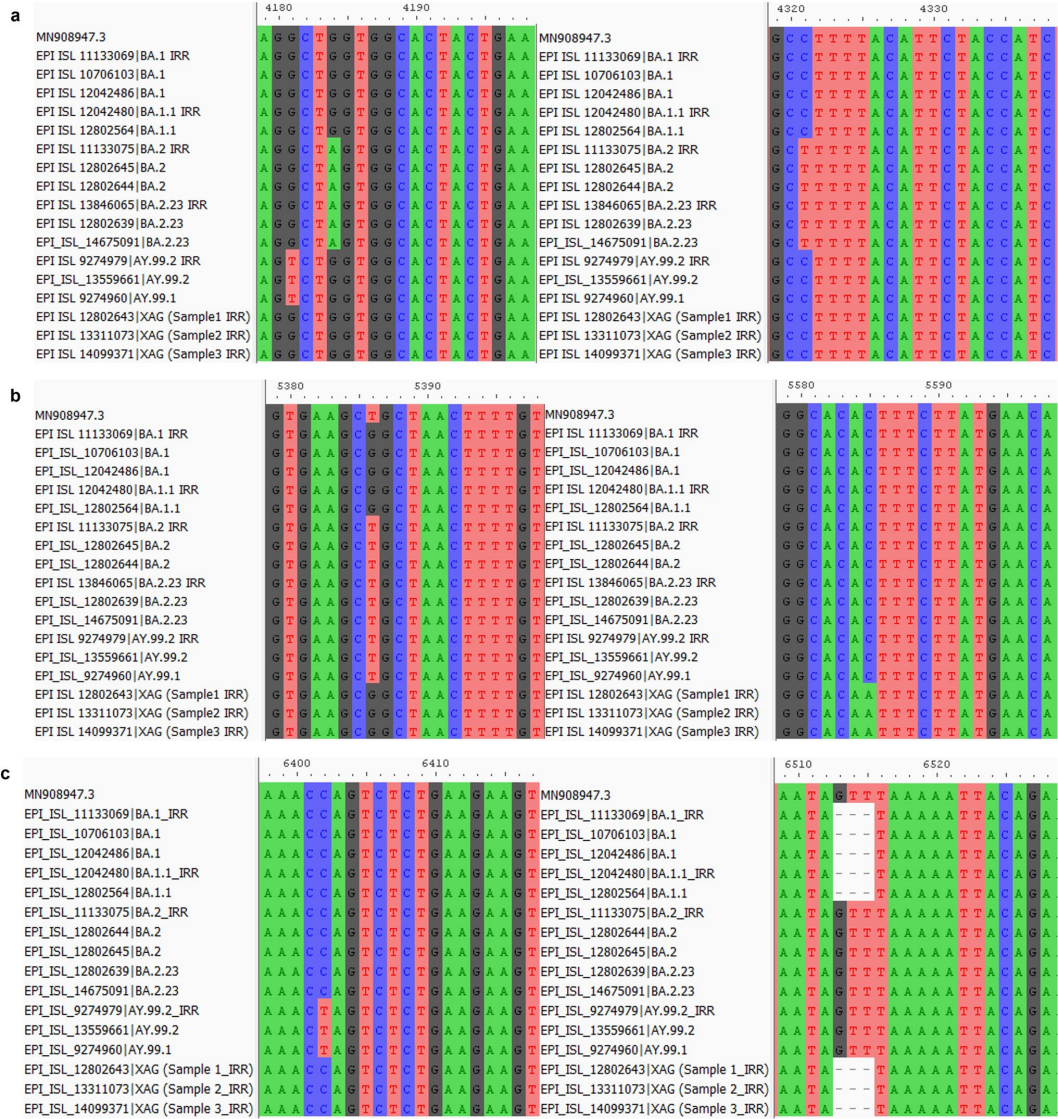
Alignment of the ORF1ab gene amplified fragments with different sets of primers. Alignments were visualized using AliView software (version 1.28). (**a**) alignment with primers 14_LEFT and 14_RIGHT; (**b**) alignment with primers 18_LEFT and 18_RIGHT; (**c**) alignment with primers 21_LEFT and 21_RIGHT. The dots represent equal nucleotides between the sequences and the reference (MN908947.3). The samples labelled IRR are samples from our study, while the samples without this denomination are samples collected from a public database—GISAID. MN908947.3: Severe Acute Respiratory Syndrome Coronavirus 2 isolate Wuhan-Hu-1, complete genome

Three regions of the ORF1ab gene, each containing one unique XAG mutation (at genome position 5585), were amplified to identify key mutations in the XAG recombinant. This allows us to distinguish between this recombinant and its parental lineages, as well as to differentiate it from Delta. The profile of mutations identified in the three amplified fragments is presented in supplementary Table 1. The results of the alignments are shown in Fig. 5. In primer set 14_LEFT and 14_RIGHT, it was possible to identify one mutation (nucleotide position G4181T) that was only present in the Delta sequences, and two mutations (nucleotide position G4184A and C4321T) that were only present in the sequences of BA.2/BA.2.23. With primer set 18_LEFT and 18_RIGHT, it was possible to distinguish between strains BA.1, BA.1.1 and XAG and the others due to the nucleotide position 5386 (T > G). In addition, it was possible to identify the exclusive mutation of the recombinant XAG (nucleotide position C5585A). With the 21_LEFT and 21_RIGHT primer sets, we did not achieve satisfactory sequencing results for the BA.2.23 sequence from our study (EPI_ISL_13846065). Despite this, it was possible to identify mutations in the Delta sequences (C6402T) and the 6512 deletion, present in the BA.1, BA.1.1 and XAG sequences. Using each of the sets of primers that identified lineage-defining mutations in the ORF1ab and Spike genes, it was possible to distinguish recombinant XAG from the BA.1, BA.1.1, BA.2, BA.2.23 and Delta lineages through analyzing the sequenced regions.

### Evaluation of the results of Sanger sequencing in comparison with NGS results

To determine the consistency of the Sanger sequencing for identifying SARS-CoV-2 variants, samples sequenced by NGS and Sanger were chosen randomly, and the results of both methodologies were assessed. For this, we compare Sanger and NGS sequencing in 227 samples (or 71,6% of the sequences). The selected samples had a mean Ct value of 23.0 ± 2.0 (E Gene) in the RT-qPCR for the detection of SARS-CoV-2 RNA. The lowest sequenced viral load is equivalent to a Ct value of 30.0. The detailed results of the sequences, including the mutations detected in each, can be seen in Supplementary Table 2. The complete genomes sequenced by the NGS method showed other mutations along the other genes that are not presented here.

**Fig. 6:**
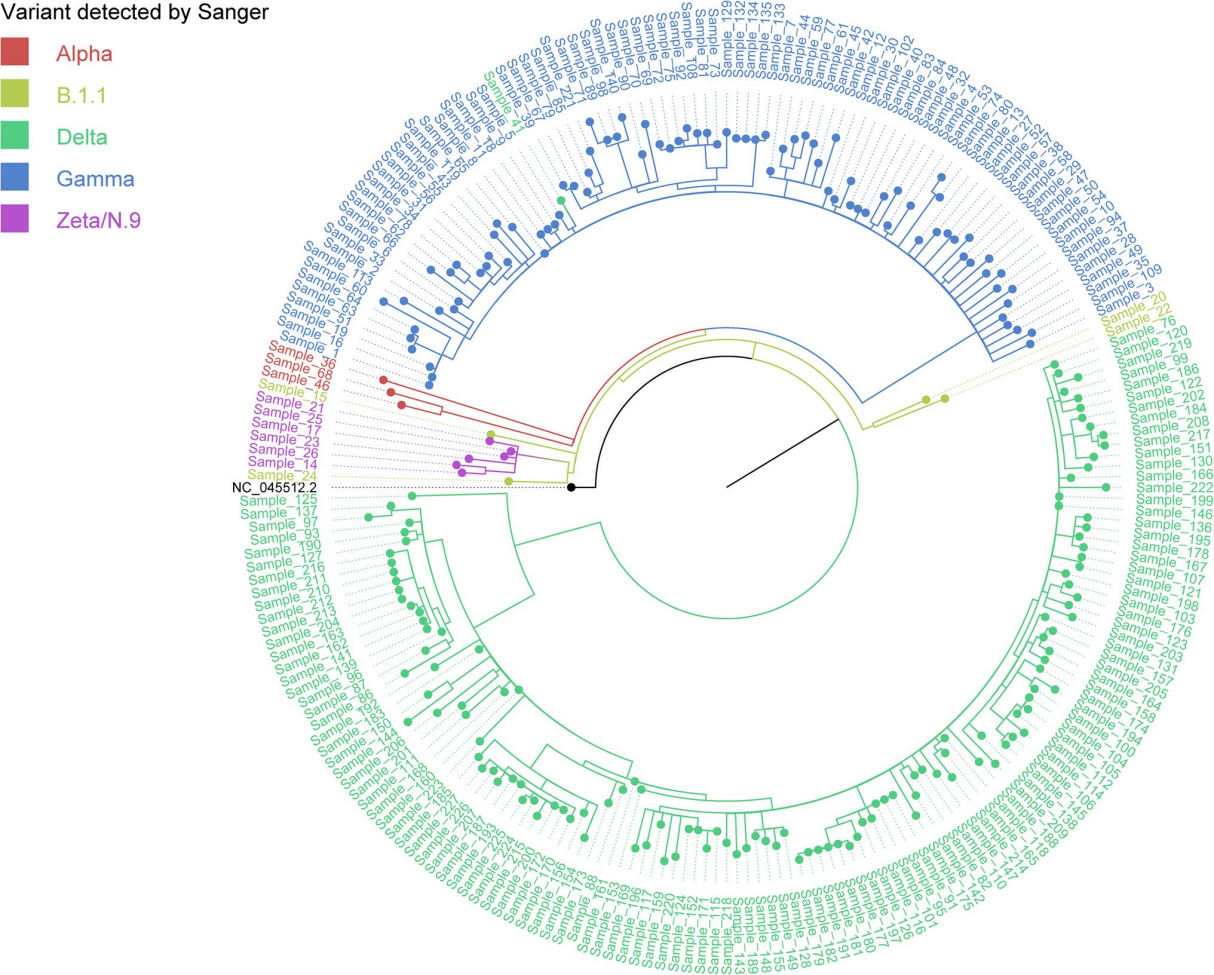
Phylogenetic tree of SARS-CoV-2 sequences from NGS. The Maximum-likelihood tree was reconstructed using IQTree (version 2.0.7). The substitution model was GTR + F + I according to ModelFinder. Only the NGS sequences were used to reconstruct this phylogenetic tree, considering the classifications obtained from Sanger as color clustering. The variants are shown with a specific color: Alpha variant (red), Gamma variant (blue), B.1.1 lineage (yellow), Delta variant (green) and Zeta/N.9 (pink). As Sample 41 was a sample from a co-infection event, it was grouped with the Gamma variant sequences. Variants are categorized by consensus sequence in NGS analyses. Therefore, the classification of this sequence as Delta could have resulted from a higher frequency of Delta mutations than Gamma mutations. NC_045512.2: Severe acute respiratory syndrome coronavirus 2 isolate Wuhan-Hu-1 (highlighted in black). The complete genome was used as a reference sequence and is the root of the tree

As expected, the NGS results were more detailed, identifying the samples at the strain level with all mutations. Nevertheless, it was demonstrated that lineage-defining mutations could be accurately identified using Sanger, and this finding was subsequently confirmed through NGS. This data validates the use of Sanger as an alternative tool to NGS for monitoring variants. Due to the more complete result of NGS, samples identified as B.1.1 in Sanger were identified as B.1.1.28 or B.1.1.33 in NGS. Despite this, the Sanger sequencing results were similar to the NGS results, demonstrating that both methodologies present comparable results. Figure 6 shows a phylogenetic tree made of SARS-CoV-2 sequences from NGS. The categorization of the samples assessed by Sanger was used to categorize the sequences. As can be observed, the sequences are grouped in the same cluster, suggesting complementarity in the results generated by both methods. In the tree, we can also observe that Sanger's Zeta and N9 sequences without definition are grouped in the same cluster, supporting the NGS finding that we only had sequences from the Zeta variant and not from the N9 lineage. Sample 41 was obtained during a co-infection scenario. After NGS sequencing, it exhibited a genetic profile reminiscent of two variants: Gamma and Delta. Interestingly, a considerable portion of mutations in this sample's sequence, found by NGS mutation analysis, aligns with the Gamma variant. However, upon conducting Sanger sequencing, specifically targeting the Spike region, a genomic sequence similar to the Delta variant was identified. In phylogenetic reconstruction, NGS-generated sequences were categorized based on results from Sanger sequencing. Consequently, despite the grouping of Sample 41 with Gamma variant sequences by NGS, even Sanger sequencing classified this sample as belonging to the Delta variant.

Sanger sequencing proved to be less expensive than NGS in terms of the price of sequencing per sample. Sanger's pre-sequencing processes (reaction, precipitation, resuspension, and plate reading protocols) cost about $4,68, (1 USD = 5,344 BLR), whereas NGS procedures (library preparation, purification, quantification, quality analyses, and the run itself) cost nine times the amount per sample. The costs associated with the maintenance of sequencing equipment are not considered, as it is the technological sequencing platforms of the Rene Rachou Institute that provide these services. However, the costs per sample are similar to other publications [11, 22]. In comparison to NGS sequencing, the runtime of Sanger was also considerably shorter. Our research group established a Sanger sequencing strategy for the samples. It took around 4 days to receive, amplify, purify, and sequence the samples. Before the NGS sequencing procedures were established, the shorter execution time of Sanger was essential for a quick response to health services.

### Assessment of the frequency and temporal distribution of SARS-CoV-2

**Fig. 7:**
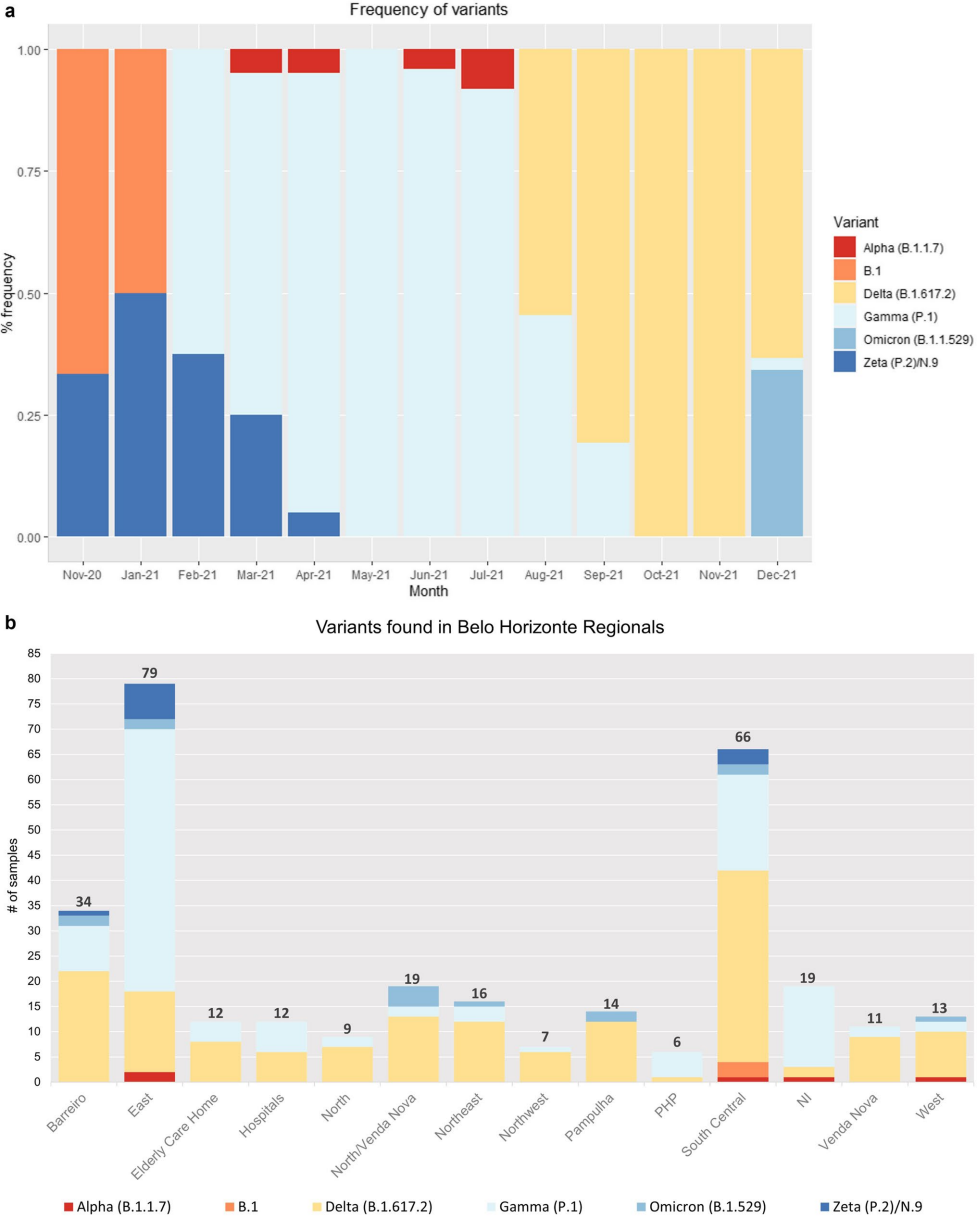
Assessment of the frequency and temporal distribution of SARS-CoV-2. The evaluations were made using the 317 sequenced samples. (**a**) represents the temporal frequency of variants found between November 2020 and December 2021. (**b**) represents the absolute number of samples and variants identified in Health Regionals of the municipality. The North/Venda Nova Unit was located at an intersection between the two Health Regionals (North and Venda Nova). No samples were collected in December 2020. PHP: Private Healthcare Professionals; NI: Not Informed

As previously mentioned, the tool presented here was used throughout 2021 to provide data quickly to epidemiological surveillance services on the known circulating variants in Belo Horizonte and the metropolitan region (Fig. 7a). As seen in Fig. 7b, samples were obtained from all Health Regionals of the municipality (Barreiro, West, South Central, Northwest, East, Northeast, Pampulha, North, and Venda Nova) to monitor the circulation of variants and adopt the necessary measures to cope with the pandemic. The North/Venda Nova Unit was located at an intersection between the two Health Regionals (North and Venda Nova). Specific groups were also analyzed, including older adults in Elderly Care Homes and hospitals, as well as healthcare professionals, due to their elevated risk of virus spread (Fig. 7b). Reports were generated weekly, enabling the generation of the graph shown in Fig. 7a. The East Regional presented more samples due to an important hospital located in this Regional. The South Central Regional had a greater number of samples than the others as it was one of the main units specialized in COVID-19 in the municipality. The Regionals show a predominance of the Gamma and Delta variants, as these were prevalent in Brazil and consequently in this study during 2021.

Between November 2020 and December 2021, the following were found: 4 samples of the Alpha variant; 4 of the B.1 lineage; 161 of the Delta variant; 123 of the Gamma variant; 14 of the Omicron variant and 11 of the Zeta/N.9 variant. The amplified region used in the Sanger method did not allow for the distinction between the Zeta and N.9 variants, as both exhibited the E484K and D614G mutations in the analyzed region. Despite this, the N.9 variant did not have significant circulation in Brazil, unlike the Zeta variant which, in addition to being considered VOI, had a peak in circulation in the early months of 2021. [23]. During the initial comparison between the Sanger and NGS methods, NGS could determine that some samples identified as Zeta/N.9 by Sanger were from the Zeta lineage. Subsequently, all samples were analyzed using the NGS method, and classified as Zeta (Supplementary Table 2). Concerning the frequency of variants identified by month, a higher frequency of Zeta variant was observed in the months of November and January, with a decline in frequency in the months of February and March of 2021. This decline of the Zeta variant coincides with the increase in the frequency of the Gamma variant in February 2021, when the Gamma variant was identified in Brazil [10, 15]. The Gamma variant appeared in Amazonas in late 2020 and soon spread throughout the state. It did not take long for the variant associated with high transmissibility to spread throughout Brazil (reaching the Southeast), and replace the Zeta variant [10]. According to our results, the Gamma variant prevailed in Belo Horizonte until August 2021, when the Delta variant was detected. In turn, Delta was the predominating variant by October 2021 (Fig. 7a). The Omicron variant was observed in the final epidemiological weeks of 2021. It is important to note that all these results were transmitted weekly to the epidemiological surveillance services to monitor VOC/VOI variants and implement control measures promptly.[24].

## Materials and methods

### Sample collection and SARS-CoV-2 RNA detection

397 nasopharyngeal and oropharyngeal swab samples were collected from November 2020 to December 2021. Samples were collected from different health centers in Belo Horizonte and its metropolitan region, in Minas Gerais state, Brazil. SARS-CoV-2 RNA detection was performed at Belo Horizonte’s Municipal Reference Laboratory using the Allplex™ SARS-CoV-2 kit (Seegene, South Korea), which targets the E, S/RdRp, and N genes of SARS-CoV-2. For some of the samples – collected at the Hospital da Baleia and Hospital Metropolitano Dr. Célio de Castro – the diagnosis was carried out at the Laboratory of Immunology for Viral Diseases at Instituto René Rachou—Fiocruz/MG through RNA extraction by the QIAamp Viral RNA kit (Qiagen, USA). The RT-qPCR was performed using an adapted Charité/Berlin WHO protocol for the detection of SARS-CoV-2 E gene [25], and the Center for Disease Control and Prevention (CDC-EUA) protocol that detects the amplification of two regions of the SARS-CoV-2 N gene (N1 and N2) [26]. This study was reviewed and approved by the Research Ethics Committee involving human beings at Instituto René Rachou, Fundação Oswaldo Cruz, under license protocol number 4084902 and CAAE (certificate of presentation for ethical appreciation): 31,984,720,300,005,091.

### Amplification, purification, sequencing, and data analysis

Positive samples with a cycle threshold (Ct) lower than 30 were amplified with a set of primers (nCoV-2019_75_LEFT/nCoV-2019_77_RIGHT) from the ARTIC Protocol [27]. This amplifies a 1.006 bp region of the SARS-CoV-2 spike protein coding sequence at subunit 1, which comprises the Receptor-binding domain (RBD). This pair of primers, together with a third internal primer (nCoV-2019_76_LEFT), were used to sequence the amplicon (Supplementary Table 3). The amplified region allows the identification of mutations at positions 417, 440, 446, 452, 477, 478, 484, 493, 496, 498, 501, 505, 547, 570 and 614. These are considered variant-defining mutations, and make it possible to identify and differentiate between VOI and VOC. RT-PCR was performed with the GoTaq® Probe 1-Step RT-qPCR System Kit (Promega, Madison, USA) according to the following protocol: 45 °C for 60 min; 95 °C for 2 min followed by 35 cycles at 95 °C for 15 s, 55 °C for 30 s, and 60 °C for 2 min. After resolving the amplicons in 1% agarose gel electrophoresis, samples were purified using the Qiagen Gel Extraction Kit (Qiagen, USA) and quantified with Qubit 1X dsDNA HS Assay (Invitrogen, ThermoFisher, Waltham, USA). Approximately 10 ng of DNA was sent for sequencing on the Sanger Capillary Sequencing Platform at Instituto René Rachou—Fiocruz/MG, using the ABI 3730 (Applied Biosystems, ThermoFisher, Waltham, USA). For Sanger data, the DNA of the samples was sent in duplicates of the primers. After sequencing, the contigs were assembled using the sangeranalyzeR package in R, with the parameter to trim at a Phred Score of 20. The generated contigs were aligned with the reference sequence to evaluate the mutation profile in the sequenced fragments. The previously described protocol was also employed to detect recombinant lineages after the identification of the recombinant XAG by our research group in samples collected in Belo Horizonte [28]. Three samples of this recombinant, designated as XAG, were also sequenced by Sanger to assess the method's ability to identify BA.1/BA.2 recombinants. For this, three sets of primers were added to the protocol to detect key XAG mutations, especially in the region of the ORF1a gene, and to the primers used to identify mutations in the spike protein (Supplementary Table 3). Eight sequences from our study were sequenced with three sets of primers of ORF1a region gene. The sequences obtained were aligned using MEGA-X (version 10.2.6) software through MUSCLE (v3.8.31), with a dataset previously assembled with nine sequences deposited in the EpiCOV™ database of GISAID (Supplementary Table 4).

Next-generation sequencing (NGS) of some samples (n = 227) was also performed, in addition to the Sanger sequencing, to identify variants and to compare the Sanger and NGS methods. The samples were sequenced using the Illumina MiSeq Platform with the Illumina DNA Prep Kit for paired-end library construction (Illumina, San Diego, USA). The samples were sequenced using the following kits: MiSeq v2 300-cycle kit was used for samples 1 to 27 and 88 to 117; MiSeq v3 600-cycle kit for samples 28 to 87; and the MiSeq v2 500-cycle kit for samples 118 to 227. The raw reads were trimmed using Trimmomatic [29] with a sliding window of four nucleotides and an average Phred score of 20. Trimmed reads smaller than 50 bp were removed. The filtered reads were mapped to the SARS-CoV-2 reference genome (NC_045512) using BWA [30] with the default parameters. The nucleotide variants were identified using iVar [31], with a minimum frequency of 50% and depth of thirty reads. The consensus sequences generated by iVar were submitted to Pangolin [32] for SARS-CoV-2 lineage identification.

## Discussion

RNA viruses such as SARS-CoV-2 accumulate genetic changes, some of which allow adaptations due to selective pressures induced by antivirals, vaccines, and host immunity [33]. The spread of variants impacts on transmission rates, diagnostic procedures, disease severity, vaccine response, and patient management [34]. From the beginning of the pandemic, the most commonly used method for identifying SARS-CoV-2 variants has been the NGS. Although it results in complete genome sequences, the NGS technique is excessively expensive both in terms of infrastructure and trained personnel. This makes it challenging to implement, especially in low-income countries. Limited access to NGS methods has led to a global disparity in SARS-CoV-2 genomic surveillance. By mid-July of 2021, more than 2 million SARS-CoV-2 genomes had been submitted to GISAID. 94% of these came from high-income countries, and only 6% from low- and middle-income countries [35].

The Sanger method has been an important tool during the pandemic for the identification and tracking of SARS-CoV-2 variants. In some cases, the methodology also focused on the sequence of the spike protein, using a smaller fragment than the one used in this work. This was still sufficient for the identification of already-known VOCs and VOIs in Tunisia in 2021 [36]. The Sanger method also makes it possible to use several fragments that target specific mutations in the spike protein gene, not just in the RBD [37–40]. Multiple reactions were used in all of these cases, and the VOCs and VOIs were efficiently identified. It is possible to use other strategies, targeting the spike protein gene and the N gene, which allows the surveillance of known variants, the monitoring of new variants, and vaccine escape mutants [18]. As with other studies, ours identified and tracked variants using the Sanger method. The difference with ours, however, was the choice of a single spike/RBD fragment that was amplified using One-Step RT-PCR, which accelerated the entire process [18]. Even after the COVID-19 pandemic, WGS is not a reality in many countries. Millions of samples need to be analyzed globally to generate important data to understand the pandemic [41].

Our work presents a rapid, accessible, and cost-effective alternative for genome sequencing, one which quickly detected the predominance of Gamma and Delta variants in the studied population, as well as the appearance of Omicron at the end of 2021. Even though NGS has made significant technological advancements and cost reductions, which have contributed to the adoption of NGS applications in clinical area, this methodology is still not widely available and presents higher costs [42]. Sanger sequencing is more affordable than NGS. The cost per sample of the Sanger reaction, precipitation, resuspension, and plate reading protocols is around $4,67 (1 USD = 5,344 BRL). The cost of performing Sanger is corroborated by other authors, such as Dorlass and Jørgensen [11, 22]. As for NGS, the cost is $42,10 per sample, including library preparation, purifications, quantifications, quality analysis and the run itself. These expenses do not account for spending related to the acquisition of equipment for sequencing, or its maintenance, or the space required on servers for data storage. In our study, both methodologies were performed using the equipment provided by the sequencing platforms of the René Rachou Institute.

When assessing costs associated with NGS, it is crucial to consider several factors, including equipment acquisition, reagents required for sequencing and library preparation, as well as costs related to data storage and analysis [43]. In our study, the average cost of reagents used for NGS totaled approximately $32,510.75. The samples were sequenced following the established process for samples received in the laboratory, using different kits for each run. Regarding the costs of the kits: for MiSeq v2 300-cycles: $1,289.00; for MiSeq v2 500-cycles: $1,450.00; for MiSeq v3 600-cycles: $1,887.00; for the COVIDSeq test: $29,846.65; and for the IDT Index Set 1–4: $1,122.10. For Sanger sequencing, the total cost of reagents was approximately $725.50, of which around $202.50 was expended with IDT primers [44] and $523.00 with GoTaq® Probe 1-Step RT-qPCR System Kit [45]. Overall, the costs associated with Sanger sequencing are eight times lower than those for NGS, resulting in a reduced cost per sample for Sanger sequencing. Regarding equipment acquisition, in 2012, the Illumina MiSeq platform cost around $128,000.00 [46]. Adjusting for inflation, in 2024, this value would correspond to approximately $125,870.00 (https://www.illumina.com/systems/sequencing-platforms/miseq.html). On the other hand, in 2020, the ABI 3730 equipment from Applied Biosystems was available for $421,000.00 (https://www.thermofisher.com/order/catalog/product/br/pt/A41046). It's essential to consider the depreciation of local currencies, particularly in low- and middle-income countries, as it can increase the costs associated with purchasing those equipments.This shows that Sanger methodology was a financial benefit when considering the costs with reagents required for sequencing procedures.

Other aspects, such as processing and analysis, must be considered. In both cases, Sanger sequencing seems to be more beneficial. Unlike NGS, Sanger sequencing offers greater flexibility when it comes to the number of samples per sequencing run. Our Sanger sequencing platform can process up to 48 samples per run. To optimize processing and maximize efficiency, we implemented a routine procedure for managing incoming samples. It took approximately four days from the time the samples were received and processed at the laboratory – which includes RNA extraction, amplification, sequencing, and analysis—to produce the report with the identification of the circulating variants. One of its advantages is that the amplification is done via a One-Step RT-PCR, which speeds up the entire process. The choice of the 1.006nt fragment of the spike gene was fundamental to its efficiency. It is possible to monitor all mutations in the RBD region. During the implementation of this sequencing tool, another fragment of the spike gene was used (~ 600nt), but it covered a smaller number of mutations, which could generate unsatisfactory results.

In our data, we observed a higher frequency of the Gamma variant in Belo Horizonte, Minas Gerais state, between March and July of 2021. The introduction of the Gamma variant in Manaus coincided with an increase in the number of cases and deaths. It was identified as one of the factors that caused an increase in the number of cases in Manaus between December 2020 and January 2021 [10] and remained dominant in circulation frequency until August 2021, when it was replaced by the Delta variant [23]. This promptly renewed concerns about the evolutionary capacity of the virus to override public health interventions and increased population immunity. In these three “501Y lineages” there were convergent mutations in other regions of the genome, which promoted the persistence of these different lineages in the face of increasing host immune recognition [24]. In the case of the Omicron variant, evidence suggests that the high transmission rate was related to immune system escape, both to currently used vaccines and through trials using sera from vaccinated and convalescent individuals [47–49]. The viral evolution profile of SARS-CoV-2 observed during the COVID-19 pandemic demonstrates the importance of the early detection of variants [50].

In almost eighty percent of the collected samples, we were able to sequence and identify the circulating variations using Sanger sequencing. The findings in approximately twenty percent of the samples with no amplification or sequencing results could be explained by the fact that these samples come from variants with a high number of mutations in the region examined in the study. This can interfere with the primers' ability to bind to the template strand, causing a failed amplification result and, consequently, a negative sequencing result. This information demonstrates the importance of monitoring SARS-CoV-2 variants, which can be optimized using different methodologies, screening samples initially by Sanger and then sequencing by NGS. Such a strategy would be more cost-effective, especially in low- and middle-income countries [18].

Through the study's methodology, our research group detected the first community transmission of the Delta variant in Belo Horizonte, Minas Gerais State, Brazil (Unpublished data). The identification occurred in samples from an outbreak at the Hospital Metropolitano Dr. Célio de Castro in Belo Horizonte, involving 25 people. At the end of July 2021 and the beginning of August 2021, fourteen professionals presented symptoms, such as fever, coughing, body pain and runny nose. Professionals infected in the outbreak were contacted and instructed to quarantine by the health services, even those without symptoms. Positive samples with Ct < 25 (SARS-CoV-2 RT-qPCR; E gene) were sequenced by Sanger and NGS, and it was possible to identify the Delta variant in 4 samples. People infected with the Delta variant in this outbreak were vaccinated with two doses of CoronaVac, applied between January and April of 2021. Early identification and quick decision-making were crucial for limiting the transmission of the Delta variant. Therefore, early identification is important to prevent new outbreaks.

Our study has some limitations, such as the difficulty in differentiating between Zeta, N.9, and Mu variants, which have the same mutation signature in the region analyzed with the primers used in the study. Despite this, the N.9 and Mu variants had no epidemiological importance in Minas Gerais [35]. An alternative developed by other research groups involves the use of a different set of primers that includes regions with variant- mutations for each of the variants, which is an advantage of Sanger. The technique can be adapted, by designing and using a new set of primers, without interfering with the protocol for identifying new variants [11, 12]. Despite the low number of samples over a short period, our data corroborate the circulation profile of variants in Brazil and in the state of Minas Gerais, which, in turn, also does not present a substantial number of sequenced genomes either. There was no surveillance by NGS in Minas Gerais state between 2020 and 2021 [23], mainly because of the high cost, a global shortage of reagents, and a lack of specialized laboratory infrastructure and well-trained staff. The sequencing of COVID-19 samples is extremely important, and as made clear by our data, the Sanger method fills the gap, as it is a fast and easy-to-perform technique.

Recombination events in RNA viruses have been described previously, especially in coronaviruses [51]. By March 2023, the Pangolin algorithm classified sixty-five recombinant strains worldwide [32]. Recombination between strains of the Omicron variant, notably the BA.1/BA.1.* and BA.2/BA.2.* strains, generated most of the identified recombinants (pango-designation/lineage_notes.txt at master · cov-lineages/pango-designation · GitHub). Recombination between Delta and Omicron variants makes up a small percentage of identified recombinants. The co-circulation of these variants favored recombination events between them [52]. In March 2024, the variant that is presently most prevalent in Brazil is JN.1. In contrast, a significant portion of the country's sequenced genomes belong to the XDR recombinant (16% of sequences), a variant resulting from the recombination of JD.1.1.1 and JN.1.1 lineages [53].

Identifying recombinants by sequencing techniques is not an easy task. It requires more detailed observation by qualified professionals, due to the similarity of the recombinant sequences with the parental lineages [54, 55]. Our research group identified a recombinant that was the recombinant with the highest circulation in Brazil between March and June of 2022 [28]. In this study, it was possible to identify the recombinant sequences by adding new primers to the already pre-established protocol for identifying the portion of the parental variants of XAG. Using this methodology to detect recombinants helps us get a better understanding of the dynamics of different viral populations that co-circulate in the same period and space. It also helps us understand the impact that recombination events have on the epidemiology of COVID-19 [51]. Furthermore, the protocol used to detect the recombinant is already standardized and effective. Our results show that Sanger sequencing can complement NGS, helping to detect recombination events quickly and affordably.

Importantly, using Sanger to monitor variants is not a replacement for NGS or any other whole genome sequencing method for identifying new variants. We propose that Sanger sequencing acts to (I) monitor the circulation of variants, complementing NGS with faster results, and (II) assist decision-making by public health agencies, helping to control the dissemination of new variants and re-infections. The Sanger-based approach is widely used to monitor a broad spectrum of viral diseases, such as the dengue virus [40], influenza [41], HIV-1 [42], hepatitis C virus (HCV) [43], yellow fever virus (YFV) [44] and even SARS-CoV-2 [11, 12, 14]. In the case of the latter, the study presented here was of great importance for providing data to the epidemiological surveillance service during the pandemic in Brazil, especially in Minas Gerais state, where NGS sequencing data were scarcer [23]. The constant changes in SARS-CoV-2 genome raises a concern about the failure to identify new variants. Considering the importance of maintaining effective genomic surveillance, early in silico analyses done by our research group indicate that the Sanger sequencing methodology, used in this work, can identify actual circulating variants, including EG.5.*, GK.1.*, JD.1, and XBB.1.*.

## Conclusions

In conclusion, our results demonstrate that the Sanger technique is an alternative to other higher-cost methods, such as NGS, for monitoring and identifying SARS-CoV-2 variants. Sanger sequencing, with its straightforward execution and quick results, could be used as an epidemiological surveillance tool, helping public health agencies to contain and control circulating variants and minimize impacts on the population. It is worth noting that the work described here identified the predominance of Gamma, the predominance of Delta, and the emergence of Omicron using the same protocol, unlike the NGS and qPCR protocols, which needed constant improvements throughout the pandemic [56]. In addition, the Sanger-based protocol can detect emerging variants, such as Omicron (B.1.1.529), and other variants yet to emerge [57].

## Supplementary Information

Below is the link to the electronic supplementary material.

Supplementary file1 The following supporting information can be downloaded at www.mdpi.com/xxx/s1: Figure S1: Electropherograms of one of the nCoV-2019_75_LEFT primer duplicates sent for sequencing of study samples, demonstrating amino acid changes (bottom of images) as well as the position of mutations in the genome (highlighted in yellow). Samples 36 (a), 106 (b), and 113 (c) are described in supplementary table 2. The Omicron sample (d) is included in the study but is not described in supplementary table 2 (JPEG 244 KB)

Supplementary file2 (PDF 43 KB)

Supplementary file3 (PDF 254 KB)

Supplementary file4 (PDF 18 KB)

Supplementary file5 (PDF 21 KB)

## Data Availability

The datasets analysed during the current study are available in The European Nucleotide Archive repository under project number PRJEB49204 and all genome sequences and associated metadata in this dataset are published in GISAID’s EpiCoV database (GISAID Identifier: EPI_SET_230713dm).
