# Supplementary figures and images for: Enhancing the epidemiological surveillance of SARS-CoV-2 using Sanger sequencing to identify circulating variants and recombinants
Source: Braz J Microbiol. 2024 May 28;55(3):2085–99. doi: 10.1007/s42770-024-01387-x (PMC11405360; doi:10.1007/s42770-024-01387-x)

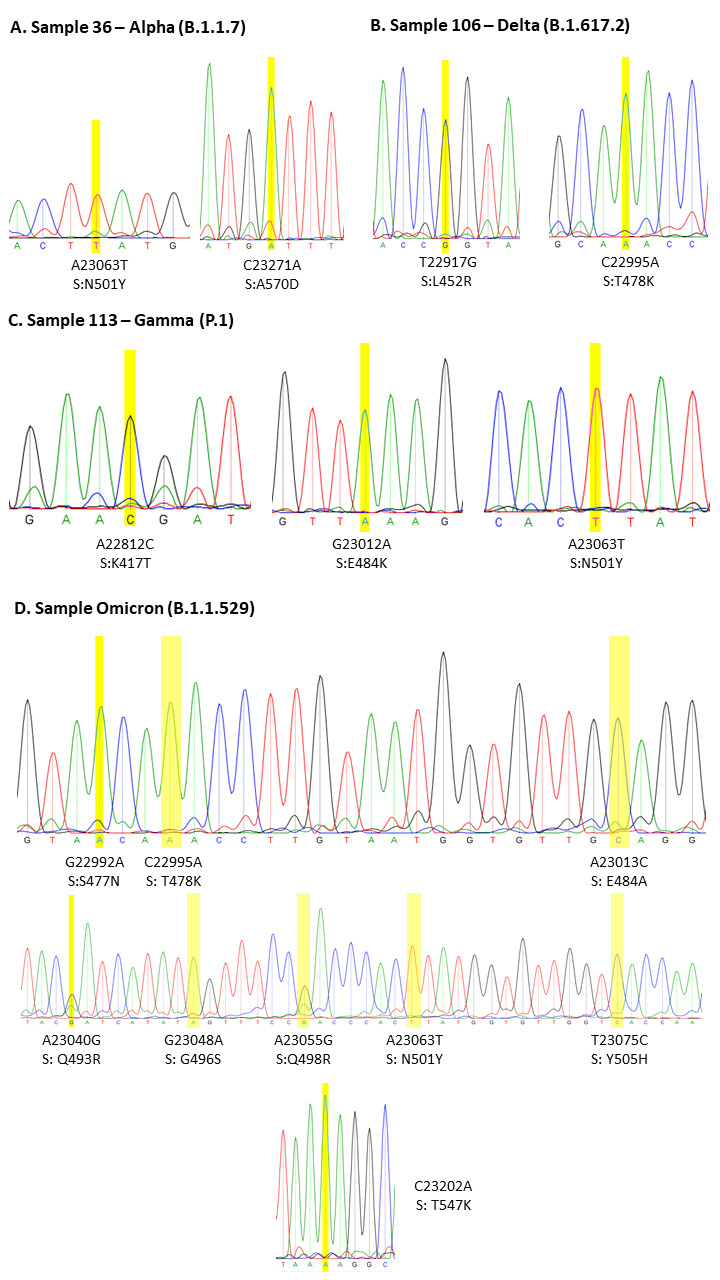

Supplement: Supplementary file 1 — Supplementary file1 The following supporting information can be downloaded at www.mdpi.com/xxx/s1: Figure S1: Electropherograms of one of the nCoV-2019_75_LEFT primer duplicates sent for sequencing of study samples, demonstrating amino acid changes (bottom of images) as well as the position of mutations in the genome (highlighted in yellow). Samples 36 (a), 106 (b), and 113 (c) are described in supplementary table 2. The Omicron sample (d) is included in the study but is not described in supplementary table 2 (JPEG 244 KB) [file 42770_2024_1387_MOESM1_ESM.jpeg]
