# Supplementary material for: Enhancing the epidemiological surveillance of SARS-CoV-2 using Sanger sequencing to identify circulating variants and recombinants
Source: Braz J Microbiol. 2024 May 28;55(3):2085–99. doi: 10.1007/s42770-024-01387-x (PMC11405360; doi:10.1007/s42770-024-01387-x)
Supplement: Supplementary file 2 — Supplementary file2 (PDF 43 KB) [file 42770_2024_1387_MOESM2_ESM.pdf]

Table S1. Mutations found in the amplified fragments for identification and distinction of recombinant XAG.

Mutations in the Spike gene region fragment sequences in BA.1.1, BA.1, BA.2, BA.2.23, Delta, and recombinant XAG lineages [1]							
**Primer set**	**Mutation (Amino acid)**	**BA.1**	**BA.1.1**	**BA.2**	**BA.2.23**	**Delta (AY.99.2)**	**XAG**
nCoV-2019_75_LEFT and nCoV-2019_77_RIGHT	K417N	P	P	P	P	-	P
	N440K	P	P	- [2]	P	-	P
	G446S	P	P	-	-	-	-
	L452R	-	-	-	-	P	-
	S477N	P	P	P	P	-	P
	T478K	P	P	P	P	P	P
	E484A	P	P	P	P	-	P
	Q493R	P	P	P	P	-	P
	G496S	P	P	-	-	-	-
	Q498R	P	P	P	P	-	P
	N501Y	P	P	P	P	-	P
	Y505H	P	P	P	P	-	P
	T547K	P	P	-	-	-	-

Mutations in the ORF1ab gene region sequences of BA.1.1, BA.1, BA.2, BA.2.23, Delta, and recombinant XAG lineages.							
**Primer set**	**Mutation [3] (Nucleotide)**	**BA.1**	**BA.1.1**	**BA.2**	**BA.2.23**	**Delta (AY.99.2)**	**XAG**
nCoV-2019_14_LEFT and nCoV-2019_14_RIGHT	4181	G	G	G	G	T	G
	4184	G	G	A	A	G	G
	4321	C	C	T	T	C	C
nCoV-2019_18_LEFT and nCoV-2019_18_RIGHT	5386	G	G	T	T	T	G
	5585	C	C	C	C	C	A
nCoV-2019_21_LEFT and nCoV-2019_21_RIGHT	6402	C	C	C	C	T	C
	Del6512	P	P	-	-	-	P

Three sequences of XAG were evaluated and the profile of mutations found in all sequences was the same. [1] The D614G mutation was identified in all samples and is not shown in the table. [2] Mutation found in two of three BA.2 sequences. [3] All position of mutations is related to the position of nucleotides in the genome. **A:** Adenine; **T:** Thymine; **C:** Cytosine; **G:** Guanine; **P:** Present; **-:** Absent.
