# Supplementary material for: Enhancing the epidemiological surveillance of SARS-CoV-2 using Sanger sequencing to identify circulating variants and recombinants
Source: Braz J Microbiol. 2024 May 28;55(3):2085–99. doi: 10.1007/s42770-024-01387-x (PMC11405360; doi:10.1007/s42770-024-01387-x)
Supplement: Supplementary file 3 — Supplementary file3 (PDF 254 KB) [file 42770_2024_1387_MOESM3_ESM.pdf]

Table S2. Results of the Sanger and NGS sequencing of 227 randomly chosen samples. The D614G mutation was not added to the table but appeared in all samples sequenced. All samples were deposited at The European Nucleotide Archive under project number PRJEB49204. Samples with Ct values of N1 and N2 were made using the CDC protocol. **NI**: Not Informed; **HB**: Hospital da Baleia; **ECU**: Emergency Care Unit; **HC UFMG**: Hospital das Clínicas of Universidade Federal de Minas Gerais; **HMDCC**: Hospital Metropolitano Doutor Célio de Castro; **ECH**: Elderly Care Home; **PHP**: Private Healthcare Professional; **NP**: Not Performed. [1] Frequency of mutations found in the Spike protein of SARS-CoV-2 in the same fragment analyzed in Sanger sequencing. The frequency described in this column is related to the overall frequency of mutations per lineage, according to Outbreak.info [53]

Sample	Collecting unit	Regional	Ct (E gene)	Ct (N gene)	Mutations detected by Sanger	Sanger Result	NGS Result	NGS sub-lineage	Mutations frequency on NGS[1]
1	NI	NI	26	20	K417T/E484K/N501Y	Gamma	Gamma		K417T: 95.7% / E484K: 95.4% / N501Y: 95.4%
2	NI	NI	22	18	K417T/E484K/N501Y	Gamma	Gamma		K417T: 95.7% / E484K: 95.4% / N501Y: 95.4%
3	NI	NI	20	17	K417T/E484K/N501Y	Gamma	Gamma		K417T: 95.7% / E484K: 95.4% / N501Y: 95.4%
4	NI	NI	19	17	K417T/E484K/N501Y	Gamma	Gamma		K417T: 95.7% / E484K: 95.4% / N501Y: 95.4%
5	NI	NI	19	17	K417T/E484K/N501Y	Gamma	Gamma		K417T: 95.7% / E484K: 95.4% / N501Y: 95.4%
6	NI	NI	22	19	K417T/E484K/N501Y	Gamma	Gamma		K417T: 95.7% / E484K: 95.4% / N501Y: 95.4%
7	NI	NI	21	15	K417T/E484K/N501Y	Gamma	Gamma		K417T: 95.7% / E484K: 95.4% / N501Y: 95.4%
8	NI	NI	27	21	K417T/E484K/N501Y	Gamma	Gamma		K417T: 95.7% / E484K: 95.4% / N501Y: 95.4%
9	NI	NI	20	22	K417T/E484K/N501Y	Gamma	Gamma	P.1.1	K417T: 93.6% / E484K: 95.4% / N501Y: 95.1%
10	NI	NI	17	19	E484K/N501Y	Gamma	Gamma		E484K: 95.4% / N501Y: 95.4%
11	NI	NI	19	20	E484K/N501Y	Gamma	Gamma		E484K: 95.4% / N501Y: 95.4%
12	NI	NI	18	19	K417T/E484K/N501Y	Gamma	Gamma		K417T: 95.7% / E484K: 95.4% / N501Y: 95.4%
13	NI	NI	29	21	E484K/N501Y	Gamma	Gamma		E484K: 95.4% / N501Y: 95.4%
14	HB	East	20	23	E484K	Zeta/N.9	Zeta		E484K: 96.6%
15	HB	East	23	NP	D614G	B.1.1	B.1.1		D614G: 98.2%
16	HB	East	25	25	E484K/N501Y	Gamma	Gamma		E484K: 95.4% / N501Y: 95.4%
17	HB	East	23	21	E484K	Zeta/N.9	Zeta		E484K: 96.6%
18	HB	East	21	23	K417T/E484K/N501Y	Gamma	Gamma		K417T: 95.7% / E484K: 95.4% / N501Y: 95.4%
19	HB	East	18	17	K417T/E484K/N501Y	Gamma	Gamma		K417T: 95.7% / E484K: 95.4% / N501Y: 95.4%
20	ECU South Central	South Central	20	N1: 19 / N2: 19	D614G	B.1.1	B.1.1.33		D614G: 99.8%
21	HC UFMG	South Central	16	N1: 14 / N2: 15	E484K	Zeta/N.9	Zeta		E484K: 96.6%
22	HC UFMG	South Central	21	NP	D614G	B.1.1	B.1.1.33		D614G: 99.8%
23	HMDCC	Barreiro	18	N1: 19 / N2: 19	E484K	Zeta/N.9	Zeta		E484K: 96.6%
24	HC UFMG	South Central	15	N1: 12 / N2: 13	D614G	B.1.1	B.1.1.28		D614G: 98.6%
25	HC UFMG	South Central	23	N1: 19 / N2: 18	E484K	Zeta/N.9	Zeta		E484K: 96.6%
26	HC UFMG	South Central	21	N1: 16 / N2: 16	E484K	Zeta/N.9	Zeta		E484K: 96.6%
27	HMDCC	Barreiro	22	N1: 21 / N2: 20	K417T/E484K/N501Y	Gamma	Gamma		K417T: 95.7% / E484K: 95.4% / N501Y: 95.4%
28	HB	East	24	25	K417T/E484K/N501Y	Gamma	Gamma		K417T: 95.7% / E484K: 95.4% / N501Y: 95.4%
29	HB	East	22	21	K417T/E484K/N501Y	Gamma	Gamma		K417T: 95.7% / E484K: 95.4% / N501Y: 95.4%
30	HB	East	22	22	K417T/E484K/N501Y	Gamma	Gamma		K417T: 95.7% / E484K: 95.4% / N501Y: 95.4%
31	HB	East	23	21	K417T/E484K/N501Y	Gamma	Gamma		K417T: 95.7% / E484K: 95.4% / N501Y: 95.4%
32	HB	East	20	19	K417T/E484K/N501Y	Gamma	Gamma		K417T: 95.7% / E484K: 95.4% / N501Y: 95.4%
33	HB	East	20	21	K417T/E484K/N501Y	Gamma	Gamma		K417T: 95.7% / E484K: 95.4% / N501Y: 95.4%
34	HB	East	20	22	K417T/E484K/N501Y	Gamma	Gamma		K417T: 95.7% / E484K: 95.4% / N501Y: 95.4%
35	HB	East	20	19	K417T/E484K/N501Y	Gamma	Gamma		K417T: 95.7% / E484K: 95.4% / N501Y: 95.4%
36	HB	East	23	22	N501Y	B.1.1	B.1.1		N501Y: 3.1%
37	HB	East	17	NP	K417T/E484K/N501Y	Gamma	Gamma		K417T: 95.7% / E484K: 95.4% / N501Y: 95.4%
38	HB	East	19	NP	K417T/E484K/N501Y	Gamma	Gamma		K417T: 95.7% / E484K: 95.4% / N501Y: 95.4%
39	HB	East	18	NP	K417T/E484K/N501Y	Gamma	Gamma	P.1.7	K417T: 97.3% / E484K: 94.7% / N501Y: 94.7%
40	HB	East	15	16	E484K/N501Y	Gamma	Gamma		E484K: 95.4% / N501Y: 95.4%
41	HB	East	19	N1: 18 / N2: 19	L452R/T478K	Delta	Delta	AY.6	L452R: 97.2% / T478K: 97.3%
42	ECU South Central	South Central	26	26	K417T/E484K/N501Y	Gamma	Gamma		K417T: 95.7% / E484K: 95.4% / N501Y: 95.4%
43	ECH	ECH	23	22	E484K/N501Y	Gamma	Gamma		E484K: 95.4% / N501Y: 95.4%
44	South Central	South Central	26	27	K417T/E484K/N501Y	Gamma	Gamma		K417T: 95.7% / E484K: 95.4% / N501Y: 95.4%
45	ECU Barreiro	Barreiro	25	25	K417T/E484K/N501Y	Gamma	Gamma		K417T: 95.7% / E484K: 95.4% / N501Y: 95.4%
46	ECU South Central	South Central	23	24	N501Y/A570D	Alpha	Alpha		N501Y: 97.6% / A570D: 99.2%
47	South Central	South Central	24	24	K417T/E484K/N501Y	Gamma	Gamma		K417T: 95.7% / E484K: 95.4% / N501Y: 95.4%
48	South Central	South Central	22	23	E484K/N501Y	Gamma	Gamma		E484K: 95.4% / N501Y: 95.4%
49	PHP	PHP	24	25	K417T/E484K/N501Y	Gamma	Gamma		K417T: 95.7% / E484K: 95.4% / N501Y: 95.4%
50	ECU Barreiro	Barreiro	24	24	E484K/N501Y	Gamma	Gamma		E484K: 95.4% / N501Y: 95.4%
51	PHP	PHP	25	25	K417T/E484K/N501Y	Gamma	Gamma		K417T: 95.7% / E484K: 95.4% / N501Y: 95.4%
52	ECU South Central	South Central	20	22	K417T/E484K/N501Y	Gamma	Gamma		K417T: 95.7% / E484K: 95.4% / N501Y: 95.4%
53	ECU South Central	South Central	23	23	K417T/E484K/N501Y	Gamma	Gamma		K417T: 95.7% / E484K: 95.4% / N501Y: 95.4%
54	ECU Barreiro	Barreiro	23	23	K417T/E484K/N501Y	Gamma	Gamma		K417T: 95.7% / E484K: 95.4% / N501Y: 95.4%
55	ECU South Central	South Central	23	24	K417T/E484K/N501Y	Gamma	Gamma		K417T: 95.7% / E484K: 95.4% / N501Y: 95.4%
56	South Central	South Central	22	22	E484K/N501Y	Gamma	Gamma		E484K: 95.4% / N501Y: 95.4%
57	South Central	South Central	22	23	K417T/E484K/N501Y	Gamma	Gamma		K417T: 95.7% / E484K: 95.4% / N501Y: 95.4%
58	PHP	PHP	21	21	E484K/N501Y	Gamma	Gamma		E484K: 95.4% / N501Y: 95.4%

Sample	Collecting unit	Regional	Ct (E gene)	Ct (N gene)	Mutations detected by Sanger	Sanger Result	NGS Result	NGS sub-lineage	Mutations frequency on NGS[1]
59	South Central	South Central	22	22	K417T/E484K/N501Y	Gamma	Gamma		K417T: 95.7% / E484K: 95.4% / N501Y: 95.4%
60	ECH	ECH	24	24	E484K/N501Y	Gamma	Gamma		E484K: 95.4% / N501Y: 95.4%
61	ECU Barreiro	Barreiro	25	24	E484K/N501Y	Gamma	Gamma		E484K: 95.4% / N501Y: 95.4%
62	South Central	South Central	23	23	K417T/E484K/N501Y	Gamma	Gamma		K417T: 95.7% / E484K: 95.4% / N501Y: 95.4%
63	ECH	ECH	21	21	K417T/E484K/N501Y	Gamma	Gamma		K417T: 95.7% / E484K: 95.4% / N501Y: 95.4%
64	ECH	ECH	21	21	K417T/E484K/N501Y	Gamma	Gamma		K417T: 95.7% / E484K: 95.4% / N501Y: 95.4%
65	ECU Barreiro	Barreiro	23	23	K417T/E484K/N501Y	Gamma	Gamma		K417T: 95.7% / E484K: 95.4% / N501Y: 95.4%
66	South Central	South Central	21	22	E484K/N501Y	Gamma	Gamma		E484K: 95.4% / N501Y: 95.4%
67	ECU Barreiro	Barreiro	22	23	E484K/N501Y	Gamma	Gamma	P.1.7	E484K: 94.7% / N501Y: 94.7%
68	ECU West	West	20	21	N501Y/A570D	Alpha	Alpha		N501Y: 97.6% / A570D: 99.2%
69	ECU South Central	South Central	21	22	K417T/E484K/N501Y	Gamma	Gamma	P.1.7	K417T: 97.3% / E484K: 94.7% / N501Y: 94.7%
70	PHP	PHP	22	23	K417T/E484K/N501Y	Gamma	Gamma	P.1.7	K417T: 97.3% / E484K: 94.7% / N501Y: 94.7%
71	South Central	South Central	22	22	K417T/E484K/N501Y	Gamma	Gamma	P.1.7	K417T: 97.3% / E484K: 94.7% / N501Y: 94.7%
72	Hospitals	Hospitals	22	23	E484K/N501Y	Gamma	Gamma	P.1.7	E484K: 94.7% / N501Y: 94.7%
73	ECU East	East	26	26	K417T/E484K/N501Y	Gamma	Gamma	P.1.7	K417T: 97.3% / E484K: 94.7% / N501Y: 94.7%
74	Hospitals	Hospitals	22	22	K417T/E484K/N501Y	Gamma	Gamma	P.1.9	K417T: 94.5% / E484K: 95.2% / N501Y: 94.9%
75	West	West	23	23	K417T/E484K/N501Y	Gamma	Gamma	P.1.7	K417T: 97.3% / E484K: 94.7% / N501Y: 94.7%
76	ECU South Central	South Central	21	22	L452R/T478K	Delta	Delta		L452R: 96.8% / T478K: 97.1%
77	PHP	PHP	22	21	K417T/E484K/N501Y	Gamma	Gamma	P.1.9	K417T: 94.5% / E484K: 95.2% / N501Y: 94.9%
78	ECU Barreiro	Barreiro	19	19	K417T/E484K/N501Y	Gamma	Gamma		K417T: 95.7% / E484K: 95.4% / N501Y: 95.4%
79	ECU East	East	23	29	K417T/E484K/N501Y	Gamma	Gamma		K417T: 95.7% / E484K: 95.4% / N501Y: 95.4%
80	South Central	South Central	22	22	K417T/E484K/N501Y	Gamma	Gamma	P.1.9	K417T: 94.5% / E484K: 95.2% / N501Y: 94.9%
81	ECU Venda Nova	Venda Nova	23	23	K417T/E484K/N501Y	Gamma	Gamma	P.1.7	K417T: 97.3% / E484K: 94.7% / N501Y: 94.7%
82	ECU Barreiro	Barreiro	19	20	L452R/T478K	Delta	Delta		L452R: 96.8% / T478K: 97.1%
83	HB	East	22	21	K417T/E484K/N501Y	Gamma	Gamma		K417T: 95.7% / E484K: 95.4% / N501Y: 95.4%
84	HB	East	23	23	K417T/E484K/N501Y	Gamma	Gamma		K417T: 95.7% / E484K: 95.4% / N501Y: 95.4%
85	HB	East	22	30	K417T/E484K/N501Y	Gamma	Gamma	P.1.7	K417T: 97.3% / E484K: 94.7% / N501Y: 94.7%
86	HMDCC	Barreiro	16	N1: 26 / N2: 27	T478K	Delta	Delta		T478K: 97.1%
87	HMDCC	Barreiro	23	N1: 22 / N2: 22	L452R/T478K	Delta	Delta	AY.6	L452R: 97.2% / T478K: 97.3%
88	ECU Venda Nova	Venda Nova	23	24	L452R/T478K	Delta	Delta	AY.99.1	L452R: 94.7% / T478K: 95.3%
89	Barreiro	Barreiro	22	21	E484K/N501Y	Gamma	Gamma		E484K: 95.4% / N501Y: 95.4%
90	ECU Northeast	Northeast	25	25	E484K/N501Y	Gamma	Gamma	P.1.7	E484K: 94.7% / N501Y: 94.7%
91	Hospitals	Hospitals	24	23	L452R/T478K	Delta	Delta	AY.99.2	L452R: 94.6% / T478K: 94.8%
92	North/Venda Nova	North/Venda Nova	25	22	K417T/E484K/N501Y	Gamma	Gamma	P.1.7	K417T: 97.3% / E484K: 94.7% / N501Y: 94.7%
93	ECU Pampulha	Pampulha	23	22	L452R/T478K	Delta	Delta	AY.6	L452R: 97.2% / T478K: 97.3%
94	ECU North	North	24	24	K417T/E484K/N501Y	Gamma	Gamma		K417T: 95.7% / E484K: 95.4% / N501Y: 95.4%
95	Barreiro	Barreiro	23	27	L452R/T478K	Delta	Delta	AY.99.2	L452R: 94.6% / T478K: 94.8%
96	North/Venda Nova	North/Venda Nova	25	25	L452R/T478K	Delta	Delta	AY.6	L452R: 97.2% / T478K: 97.3%
97	ECU North	North	21	22	L452R/T478K	Delta	Delta	AY.43	L452R: 96.9% / T478K: 97.5%
98	ECU East	East	20	21	K417T/E484K/N501Y	Gamma	Gamma		K417T: 95.7% / E484K: 95.4% / N501Y: 95.4%
99	North/Venda Nova	North/Venda Nova	23	22	L452R/T478K	Delta	Delta	AY.99.2	L452R: 94.6% / T478K: 94.8%
100	North/Venda Nova	North/Venda Nova	26	26	L452R/T478K	Delta	Delta	AY.99.2	L452R: 94.6% / T478K: 94.8%
101	North/Venda Nova	North/Venda Nova	24	24	L452R/T478K	Delta	Delta	AY.99.2	L452R: 94.6% / T478K: 94.8%
102	ECU Barreiro	Barreiro	24	22	K417T/E484K/N501Y	Gamma	Gamma		K417T: 95.7% / E484K: 95.4% / N501Y: 95.4%
103	ECU North	North	21	21	L452R/T478K	Delta	Delta	AY.99.2	L452R: 94.6% / T478K: 94.8%
104	ECH	ECH	24	24	L452R/T478K	Delta	Delta	AY.99.2	L452R: 94.6% / T478K: 94.8%
105	ECH	ECH	25	24	L452R/T478K	Delta	Delta	AY.99.2	L452R: 94.6% / T478K: 94.8%
106	ECU Pampulha	Pampulha	25	26	L452R/T478K	Delta	Delta	AY.99.2	L452R: 94.6% / T478K: 94.8%
107	NI	NI	NI	NI	L452R/T478K	Delta	Delta	AY.99.2	L452R: 94.6% / T478K: 94.8%
108	ECU Venda Nova	Venda Nova	20	20	E484K/N501Y	Gamma	Gamma	P.1.7	E484K: 94.7% / N501Y: 94.7%
109	Northeast	Northeast	26	25	E484K/N501Y	Gamma	Gamma		E484K: 95.4% / N501Y: 95.4%
110	Barreiro	Barreiro	24	24	L452R/T478K	Delta	Delta	AY.99.2	L452R: 94.6% / T478K: 94.8%
111	ECU Northeast	Northeast	25	25	L452R/T478K	Delta	Delta	AY.46.3	L452R: 90.2% / T478K: 89.4%
112	ECH	ECH	23	23	L452R/T478K	Delta	Delta	AY.99.2	L452R: 94.6% / T478K: 94.8%
113	Northwest	Northwest	22	21	K417T/E484K/N501Y	Gamma	Gamma		K417T: 95.7% / E484K: 95.4% / N501Y: 95.4%
114	West	West	23	24	L452R/T478K	Delta	Delta	AY.99.2	L452R: 94.6% / T478K: 94.8%
115	ECU Pampulha	Pampulha	25	25	L452R/T478K	Delta	Delta	AY.99.2	L452R: 94.6% / T478K: 94.8%
116	ECU Venda Nova	Venda Nova	21	21	L452R/T478K	Delta	Delta	AY.99.2	L452R: 94.6% / T478K: 94.8%

Sample	Collecting unit	Regional	Ct (E gene)	Ct (N gene)	Mutations detected by Sanger	Sanger Result	NGS Result	NGS sub-lineage	Mutations frequency on NGS[1]
117	West	West	25	25	L452R/T478K	Delta	Delta	AY.99.2	L452R: 94.6% / T478K: 94.8%
118	Barreiro	Barreiro	25	25	L452R/T478K	Delta	Delta	AY.99.2	L452R: 94.6% / T478K: 94.8%
119	ECU Cachoeirinha	Northeast	27	26	K417T/E484K/N501Y	Gamma	Gamma		K417T: 95.7% / E484K: 95.4% / N501Y: 95.4%
120	ECU South Central	South Central	23	24	L452R/T478K	Delta	Delta	AY.99.2	L452R: 94.6% / T478K: 94.8%
121	ECU South Central	South Central	24	24	L452R/T478K	Delta	Delta	AY.99.2	L452R: 94.6% / T478K: 94.8%
122	Pampulha	Pampulha	25	25	L452R/T478K	Delta	Delta	AY.99.2	L452R: 94.6% / T478K: 94.8%
123	ECU Northeast	Northeast	25	25	L452R/T478K	Delta	Delta	AY.99.2	L452R: 94.6% / T478K: 94.8%
124	ECU South Central	South Central	22	21	L452R/T478K	Delta	Delta	AY.99.2	L452R: 94.6% / T478K: 94.8%
125	West	West	25	25	L452R/T478K	Delta	Delta	AY.26	L452R: 96.4% / T478K: 96.6%
126	ECU Cachoeirinha	Northeast	24	25	L452R/T478K	Delta	Delta	AY.99.2	L452R: 94.6% / T478K: 94.8%
127	ECU North	North	21	21	L452R/T478K	Delta	Delta	AY.6	L452R: 97.2% / T478K: 97.3%
128	ECU Venda Nova	Venda Nova	25	25	L452R/T478K	Delta	Delta	AY.99.2	L452R: 94.6% / T478K: 94.8%
129	West	West	24	24	K417T/E484K/N501Y	Gamma	Gamma	P.1.7	K417T: 97.3% / E484K: 94.7% / N501Y: 94.7%
130	West	West	24	25	L452R/T478K	Delta	Delta	AY.99.2	L452R: 94.6% / T478K: 94.8%
131	East	East	20	20	L452R/T478K	Delta	Delta	AY.99.2	L452R: 94.6% / T478K: 94.8%
132	Hospitals	Hospitals	27	26	E484K/N501Y	Gamma	Gamma	P.1.6	E484K: 98.9% / N501Y: 99.1%
133	Hospitals	Hospitals	25	25	K417T/E484K/N501Y	Gamma	Gamma	P.1.6	K417T: 100% / E484K: 98.9% / N501Y: 99.1%
134	Hospitals	Hospitals	25	25	E484K/N501Y	Gamma	Gamma	P.1.6	E484K: 98.9% / N501Y: 99.1%
135	Hospitals	Hospitals	24	24	K417T/E484K/N501Y	Gamma	Gamma	P.1.6	K417T: 100% / E484K: 98.9% / N501Y: 99.1%
136	West	West	22	22	L452R/T478K	Delta	Delta	AY.99.2	L452R: 94.6% / T478K: 94.8%
137	East	East	25	23	L452R/T478K	Delta	Delta	AY.6	L452R: 97.2% / T478K: 97.3%
138	ECU Pampulha	Pampulha	26	26	L452R/T478K	Delta	Delta	AY.99.2	L452R: 94.6% / T478K: 94.8%
139	ECU South Central	South Central	22	23	L452R/T478K	Delta	Delta	AY.6	L452R: 97.2% / T478K: 97.3%
140	ECU South Central	South Central	21	21	E484K/N501Y	Gamma	Gamma		E484K: 95.4% / N501Y: 95.4%
141	ECU South Central	South Central	22	23	L452R/T478K	Delta	Delta	AY.6	L452R: 97.2% / T478K: 97.3%
142	South Central	South Central	25	26	L452R/T478K	Delta	Delta	AY.99.2	L452R: 94.6% / T478K: 94.8%
143	ECU Northeast	Northeast	22	22	L452R/T478K	Delta	Delta	AY.99.2	L452R: 94.6% / T478K: 94.8%
144	PHP	PHP	22	23	L452R/T478K	Delta	Delta	AY.34.1.1	L452R:94.6% / T478K: 94.9%
145	ECU South Central	South Central	25	25	L452R/T478K	Delta	Delta	AY.99.2	L452R: 94.6% / T478K: 94.8%
146	Northwest	Northwest	26	26	L452R/T478K	Delta	Delta	AY.99.2	L452R: 94.6% / T478K: 94.8%
147	Northwest	Northwest	22	23	L452R/T478K	Delta	Delta	AY.99.2	L452R: 94.6% / T478K: 94.8%
148	ECU South Central	South Central	24	24	L452R/T478K	Delta	Delta	AY.99.2	L452R: 94.6% / T478K: 94.8%
149	ECU Northeast	Northeast	21	23	L452R/T478K	Delta	Delta	AY.99.2	L452R: 94.6% / T478K: 94.8%
150	Pampulha	Pampulha	26	28	L452R/T478K	Delta	Delta	AY.46.6	L452R: 97.4% / T478K: 97.7%
151	West	West	27	25	L452R/T478K	Delta	Delta	AY.99.2	L452R: 94.6% / T478K: 94.8%
152	NI	NI	24	25	L452R/T478K	Delta	Delta	AY.99.2	L452R: 94.6% / T478K: 94.8%
153	ECU Barreiro	Barreiro	27	26	L452R/T478K	Delta	Delta	AY.99.2	L452R: 94.6% / T478K: 94.8%
154	East	East	24	25	L452R/T478K	Delta	Delta	AY.99.1	L452R: 94.7% / T478K: 95.3%
155	North/Venda Nova	North/Venda Nova	27	26	L452R/T478K	Delta	Delta	AY.99.2	L452R: 94.6% / T478K: 94.8%
156	ECU East	East	22	22	L452R/T478K	Delta	Delta	AY.99.1	L452R: 94.7% / T478K: 95.3%
157	ECU Northeast	Northeast	23	21	L452R/T478K	Delta	Delta	AY.99.2	L452R: 94.6% / T478K: 94.8%
158	ECU Barreiro	Barreiro	24	25	L452R/T478K	Delta	Delta	AY.99.2	L452R: 94.6% / T478K: 94.8%
159	Barreiro	Barreiro	20	22	L452R/T478K	Delta	Delta	AY.99.2	L452R: 94.6% / T478K: 94.8%
160	ECU West	West	26	26	L452R/T478K	Delta	Delta	AY.46.6	L452R: 97.4% / T478K: 97.7%
161	ECU Northeast	Northeast	22	23	L452R/T478K	Delta	Delta	AY.99	L452R: 84.5% / T478K: 85.8%
162	ECU Pampulha	Pampulha	25	25	L452R/T478K	Delta	Delta	AY.6	L452R: 97.2% / T478K: 97.3%
163	ECU Pampulha	Pampulha	26	25	L452R/T478K	Delta	Delta	AY.6	L452R: 97.2% / T478K: 97.3%
164	ECU South Central	South Central	23	23	L452R/T478K	Delta	Delta	AY.99.2	L452R: 94.6% / T478K: 94.8%
165	North/Venda Nova	North/Venda Nova	24	22	L452R/T478K	Delta	Delta	AY.99.2	L452R: 94.6% / T478K: 94.8%
166	South Central	South Central	21	21	L452R/T478K	Delta	Delta	AY.99.2	L452R: 94.6% / T478K: 94.8%
167	Barreiro	Barreiro	25	26	L452R/T478K	Delta	Delta	AY.99.2	L452R: 94.6% / T478K: 94.8%
168	North/Venda Nova	North/Venda Nova	26	27	L452R/T478K	Delta	Delta	AY.114	L452R: 96.4% / T478K: 96.7%
169	ECU Venda Nova	Venda Nova	19	19	L452R/T478K	Delta	Delta	AY.99.2	L452R: 94.6% / T478K: 94.8%
170	Hospitals	Hospitals	26	26	L452R/T478K	Delta	Delta	AY.99.1	L452R: 94.7% / T478K: 95.3%
171	Northwest	Northwest	22	21	L452R/T478K	Delta	Delta	AY.99.2	L452R: 94.6% / T478K: 94.8%
172	Hospitals	Hospitals	22	22	L452R/T478K	Delta	Delta	AY.99.1	L452R: 94.7% / T478K: 95.3%
173	ECU South Central	South Central	24	23	L452R/T478K	Delta	Delta	AY.99.1	L452R: 94.7% / T478K: 95.3%
174	ECU East	East	21	22	L452R/T478K	Delta	Delta	AY.99.2	L452R: 94.6% / T478K: 94.8%

Sample	Collecting unit	Regional	Ct (E gene)	Ct (N gene)	Mutations detected by Sanger	Sanger Result	NGS Result	NGS sub-lineage	Mutations frequency on NGS[1]
175	ECU South Central	South Central	25	25	L452R/T478K	Delta	Delta	AY.99.2	L452R: 94.6% / T478K: 94.8%
176	ECU South Central	South Central	25	25	L452R/T478K	Delta	Delta	AY.99.2	L452R: 94.6% / T478K: 94.8%
177	ECH	ECH	21	21	L452R/T478K	Delta	Delta	AY.99.2	L452R: 94.6% / T478K: 94.8%
178	Barreiro	Barreiro	22	21	L452R/T478K	Delta	Delta	AY.99.2	L452R: 94.6% / T478K: 94.8%
179	ECH	ECH	21	21	L452R/T478K	Delta	Delta	AY.99.2	L452R: 94.6% / T478K: 94.8%
180	ECH	ECH	20	20	L452R/T478K	Delta	Delta	AY.99.2	L452R: 94.6% / T478K: 94.8%
181	ECH	ECH	20	20	L452R/T478K	Delta	Delta	AY.99.2	L452R: 94.6% / T478K: 94.8%
182	ECH	ECH	22	23	L452R/T478K	Delta	Delta	AY.99.2	L452R: 94.6% / T478K: 94.8%
183	ECU Venda Nova	Venda Nova	27	26	L452R/T478K	Delta	Delta	AY.46.6	L452R: 97.4% / T478K: 97.7%
184	ECU South Central	South Central	21	21	L452R/T478K	Delta	Delta	AY.99.2	L452R: 94.6% / T478K: 94.8%
185	Northwest	Northwest	25	26	L452R/T478K	Delta	Delta	AY.99.1	L452R: 94.7% / T478K: 95.3%
186	Barreiro	Barreiro	23	23	L452R/T478K	Delta	Delta	AY.99.2	L452R: 94.6% / T478K: 94.8%
187	ECU South Central	South Central	24	23	L452R/T478K	Delta	Delta	AY.99.1	L452R: 94.7% / T478K: 95.3%
188	ECU Barreiro	Barreiro	21	22	L452R/T478K	Delta	Delta	AY.99.2	L452R: 94.6% / T478K: 94.8%
189	ECU South Central	South Central	23	23	L452R/T478K	Delta	Delta	AY.99.2	L452R: 94.6% / T478K: 94.8%
190	Northwest	Northwest	25	24	L452R/T478K	Delta	Delta	AY.6	L452R: 97.2% / T478K: 97.3%
191	ECU Barreiro	Barreiro	23	22	L452R/T478K	Delta	Delta	AY.99.2	L452R: 94.6% / T478K: 94.8%
192	Northwest	Northwest	23	25	L452R/T478K	Delta	Delta	AY.3	L452R: 96.6% / T478K: 96.9%
193	ECU South Central	South Central	26	25	L452R/T478K	Delta	Delta	AY.99.1	L452R: 94.7% / T478K: 95.3%
194	ECU North	North	25	27	L452R/T478K	Delta	Delta	AY.99.2	L452R: 94.6% / T478K: 94.8%
195	ECU South Central	South Central	22	22	L452R/T478K	Delta	Delta	AY.99.2	L452R: 94.6% / T478K: 94.8%
196	ECU South Central	South Central	24	25	L452R/T478K	Delta	Delta	AY.99.2	L452R: 94.6% / T478K: 94.8%
197	ECU Barreiro	Barreiro	25	25	L452R/T478K	Delta	Delta	AY.99.2	L452R: 94.6% / T478K: 94.8%
198	ECU South Central	South Central	24	24	L452R/T478K	Delta	Delta	AY.99.2	L452R: 94.6% / T478K: 94.8%
199	ECU South Central	South Central	25	25	L452R/T478K	Delta	Delta	AY.99.2	L452R: 94.6% / T478K: 94.8%
200	ECU South Central	South Central	27	26	L452R/T478K	Delta	Delta	AY.99.1	L452R: 94.7% / T478K: 95.3%
201	ECU South Central	South Central	24	24	L452R/T478K	Delta	Delta	AY.43.1	L452R: 94.3% / T478K: 93.4%
202	ECU East	East	21	21	L452R/T478K	Delta	Delta	AY.99.2	L452R: 94.6% / T478K: 94.8%
203	ECU Pampulha	Pampulha	27	27	L452R/T478K	Delta	Delta	AY.99.2	L452R: 94.6% / T478K: 94.8%
204	ECU Northeast	Northeast	21	22	L452R/T478K	Delta	Delta	AY.6	L452R: 97.2% / T478K: 97.3%
205	ECU Pampulha	Pampulha	23	23	L452R/T478K	Delta	Delta	AY.99.2	L452R: 94.6% / T478K: 94.8%
206	ECU South Central	South Central	26	25	L452R/T478K	Delta	Delta	AY.4	L452R: 97.8% / T478K: 97.9%
207	ECU South Central	South Central	22	22	L452R/T478K	Delta	Delta	AY.99.1	L452R: 94.7% / T478K: 95.3%
208	North/Venda Nova	North/Venda Nova	23	23	L452R/T478K	Delta	Delta	AY.99.2	L452R: 94.6% / T478K: 94.8%
209	North/Venda Nova	North/Venda Nova	27	27	L452R/T478K	Delta	Delta	AY.99.2	L452R: 94.6% / T478K: 94.8%
210	North/Venda Nova	North/Venda Nova	25	24	L452R/T478K	Delta	Delta	AY.6	L452R: 97.2% / T478K: 97.3%
211	Northeast	Northeast	24	24	L452R/T478K	Delta	Delta	AY.6	L452R: 97.2% / T478K: 97.3%
212	North/Venda Nova	North/Venda Nova	27	27	L452R/T478K	Delta	Delta	AY.6	L452R: 97.2% / T478K: 97.3%
213	North/Venda Nova	North/Venda Nova	22	22	L452R/T478K	Delta	Delta	AY.6	L452R: 97.2% / T478K: 97.3%
214	ECU Venda Nova	Venda Nova	23	24	L452R/T478K	Delta	Delta	AY.99.2	L452R: 94.6% / T478K: 94.8%
215	ECU Venda Nova	Venda Nova	21	21	L452R/T478K	Delta	Delta	AY.99.1	L452R: 94.7% / T478K: 95.3%
216	North/Venda Nova	North/Venda Nova	27	27	L452R/T478K	Delta	Delta	AY.6	L452R: 97.2% / T478K: 97.3%
217	West	West	23	24	L452R/T478K	Delta	Delta	AY.99.2	L452R: 94.6% / T478K: 94.8%
218	ECU East	East	25	25	L452R/T478K	Delta	Delta	AY.99.2	L452R: 94.6% / T478K: 94.8%
219	ECU South Central	South Central	25	24	L452R/T478K	Delta	Delta	AY.99.2	L452R: 94.6% / T478K: 94.8%
220	ECU South Central	South Central	21	20	L452R/T478K	Delta	Delta	AY.99.2	L452R: 94.6% / T478K: 94.8%
221	ECU South Central	South Central	23	27	E484K/N501Y	Gamma	Gamma	P.1.7	E484K: 94.7% / N501Y: 94.7%
222	ECU Venda Nova	Venda Nova	24	22	L452R/T478K	Delta	Delta	AY.99.2	L452R: 94.6% / T478K: 94.8%
223	ECU East	East	21	22	L452R/T478K	Delta	Delta	AY.122	L452R: 95.8% / T478K: 96.3%
224	ECU South Central	South Central	21	21	L452R/T478K	Delta	Delta	AY.99.1	L452R: 94.7% / T478K: 95.3%
225	ECU South Central	South Central	30	29	L452R/T478K	Delta	Delta	AY.99.1	L452R: 94.7% / T478K: 95.3%
226	ECU South Central	South Central	24	24	L452R/T478K	Delta	Delta	AY.99.1	L452R: 94.7% / T478K: 95.3%
227	ECU North	North	25	23	L452R/T478K	Delta	Delta	AY.99.1	L452R: 94.7% / T478K: 95.3%
