# Supplementary material for: Enhancing the epidemiological surveillance of SARS-CoV-2 using Sanger sequencing to identify circulating variants and recombinants
Source: Braz J Microbiol. 2024 May 28;55(3):2085–99. doi: 10.1007/s42770-024-01387-x (PMC11405360; doi:10.1007/s42770-024-01387-x)
Supplement: Supplementary file 4 — Supplementary file4 (PDF 18 KB) [file 42770_2024_1387_MOESM4_ESM.pdf]

Table S3: Primers used in Sanger amplification and sequencing protocols to identify VOI, VOC and recombinant XAG.

Primers for amplification of a region of the Spike protein gene and Sanger sequencing for identification of VOI and VOC circulating [1]					
**Primer name**	**Sequence**	**Start**	**End**	**Amplicon size**	**Application**
nCoV-2019_75_LEFT	AGAGTCCAACCAACAGAATCTATTGT	22516	22542		Amplification and sequencing
nCoV-2019_76_LEFT	AGGGCAAACTGGAAAGATTGCT	22797	22819	1006 bp	Sequencing only
nCoV-2019_77_RIGHT	CAGCCCCTATTAAACAGCCTGC	23500	23522		Amplification and sequencing
**Primers used for amplification and Sanger sequencing for the detection of XAG recombinant [2]**					
**Primer name**	**Sequence**	**Start**	**End**	**Amplicon size**	**Application**
nCoV-2019_14_LEFT	TGGAAGAAACTAAGTTCCTCACAGAA	3992	4018	417 bp	Amplification and sequencing
nCoV-2019_14_RIGHT	CATGTGCAAGCATTTCTCGCAA	4387	4409		Amplification and sequencing
nCoV-2019_18_LEFT	TGGAAATACCCACAAGTTAATGGTTTAAC	5230	5259	413 bp	Amplification and sequencing
nCoV-2019_18_RIGHT	GCTTGTTTACCACACGTACAAGG	5620	5643		Amplification and sequencing
nCoV-2019_21_LEFT	CACTACACACCCTCTTTTAAGAAAGG	6184	6210	398 bp	Amplification and sequencing
nCoV-2019_21_RIGHT	GTAAGACTAGAATTGTCTACATAAGCAGC	6553	6582		Amplification and sequencing

MN908947.3 - Severe Acute Respiratory Syndrome Coronavirus 2 isolate Wuhan-Hu-1, complete genome. [1]The external primers used in PCR amplify a 1.006 bp sequence of the spike protein of SARS-CoV-2; [2] The primers amplify a fragment of the ORF1a gene of SARS-CoV-2.
