# Supplementary material for: Enhancing the epidemiological surveillance of SARS-CoV-2 using Sanger sequencing to identify circulating variants and recombinants
Source: Braz J Microbiol. 2024 May 28;55(3):2085–99. doi: 10.1007/s42770-024-01387-x (PMC11405360; doi:10.1007/s42770-024-01387-x)
Supplement: Supplementary file 5 — Supplementary file5 (PDF 21 KB) [file 42770_2024_1387_MOESM5_ESM.pdf]

# SUPPLEMENTAL TABLE

**Data Availability**

GISAID Identifier: EPI_SET_230713dm
doi: 10.55876/gis8.230713dm

All genome sequences and associated metadata in this dataset are published in GISAID's EpiCoV database. To view the contributors of each individual sequence with details such as accession number, Virus name, Collection date, Originating Lab and Submitting Lab and the list of Authors, visit 10.55876/gis8.230713dm

**Data Snapshot**

- EPI_SET_230713dm is composed of 17 individual genome sequences.
- The collection dates range from 2021-12-13 to 2022-06-20;
- Data were collected in 1 countries and territories;
- All sequences in this dataset are compared relative to hCoV-19/Wuhan/WIV04/2019 (WIV04), the official reference sequence employed by GISAID (EPI_ISL_402124). Learn more at https://gisaid.org/WIV04.
